# MicroRNA in Teleost Fish

**DOI:** 10.1093/gbe/evu151

**Published:** 2014-07-22

**Authors:** Teshome Tilahun Bizuayehu, Igor Babiak

**Affiliations:** Faculty of Aquaculture and Biosciences, University of Nordland, Bodø, Norway

**Keywords:** development, microRNA, organogenesis, posttranscriptional regulation, teleosts

## Abstract

MicroRNAs (miRNAs) are transcriptional and posttranscriptional regulators involved in nearly all known biological processes in distant eukaryotic clades. Their discovery and functional characterization have broadened our understanding of biological regulatory mechanisms in animals and plants. They show both evolutionary conserved and unique features across Metazoa. Here, we present the current status of the knowledge about the role of miRNA in development, growth, and physiology of teleost fishes, in comparison to other vertebrates. Infraclass Teleostei is the most abundant group among vertebrate lineage. Fish are an important component of aquatic ecosystems and human life, being the prolific source of animal proteins worldwide and a vertebrate model for biomedical research. We review miRNA biogenesis, regulation, modifications, and mechanisms of action. Specific sections are devoted to the role of miRNA in teleost development, organogenesis, tissue differentiation, growth, regeneration, reproduction, endocrine system, and responses to environmental stimuli. Each section discusses gaps in the current knowledge and pinpoints the future directions of research on miRNA in teleosts.

## Introduction

Small nonprotein-coding RNAs (ncRNAs) are short, 18–40 nucleotide (nt) sequences, with diverse biogenesis pathways and regulatory mechanisms. They have convoluted relationships, in which they cooperate, compete, or regulate each other (Ghildiyal and Zamore 2009). They are involved in basic cellular processes, including differentiation, proliferation, and apoptosis (Bartel 2009).

Among several types of small RNAs, microRNAs (miRNAs), approximately 22-nt-long posttranscriptional regulators of mRNA, have been intensively investigated in recent years. They were discovered in early 1990s (Lee et al. 1993; Wightman et al. 1993), but it took almost a decade to discover how massive their involvement in gene expression regulatory networks is. It is estimated that in mammals over 60% of mRNAs have conserved miRNA-binding sites (Friedman et al. 2009; Guo et al. 2010). Also in teleost fishes, miRNAs are involved in the development and various physiological processes (Wienholds et al. 2005; Salem et al. 2010; Bizuayehu et al. 2012a; Mishima 2012; Wei et al. 2012). Although no estimation or quantification has been made yet to determine the extent of miRNA participation in regulatory network in teleosts, it can be anticipated that a considerable set of mRNAs is under their modulation, based on high conservation of miRNA among animals in general and vertebrates in particular. Teleost miRNAs were first reported in zebrafish (Lim et al. 2003) and miRNA repositories during zebrafish development, and some functions were characterized (Chen et al. 2005; Giraldez et al. 2005; Wienholds et al. 2003, 2005). Although a number of studies on miRNA in some other teleosts has been reported since then (Salem et al. 2010; Fu et al. 2011; Xia et al. 2011; Barozai 2012; Andreassen et al. 2013; Bekaert et al. 2013; Xu et al. 2013), information on the functions of miRNAs in teleosts has largely been obtained from studies carried out on zebrafish.

Teleosts are the most speciose among the vertebrate lineage with an estimated number of species exceeding 25,000 (Nelson 2006); therefore, miRNA characterization and functional studies performed in few species so far are presumptuous to conclude on general principles. Currently, there are 1,250 miRNAs identified in 8 teleost species, representing 5 orders (miRBase v. 20), which reflects how little has been done in characterization of miRNAs in fishes when compared with mammals (table 1).

**Table 1:** Number of Precursor and Mature miRNAs Identified in Teleost Fish and Compared with Other Vertebrates (miRBase v.20)

	Precursor	Mature
Fish	1,250	1,044
*Cyprinus carpio*	134	146
*Danio rerio*	346	255
*Fugu rubripes*	129	108
*Hippoglossus hippoglossus*	40	37
*Ictalurus punctatus*	281	205
*Oryzias latipes*	168	146
*Paralichthys olivaceus*	20	38
*Tetraodon nigroviridis*	132	109
Amphibians	211	196
Reptiles	282	416
Birds	980	1,330
Mammals	9,076	11,717

Fish are important in broad terms of ecology and food production. Both exploitation and conservation tasks need a baseline knowledge of the habitat and physiology of a species in question. Understanding molecular mechanisms and functions can provide sustainable and more efficient, knowledge-based solutions. Teleosts also serve as biological models. Genetic studies in teleosts have widened our knowledge of number of biological pathways that are common in vertebrates (Howe et al. 2013). Teleosts show a number of advantages, including high fecundity, oviparity, quick development, easiness of manipulation and production of genetic modifications, early development of functional systems, and the potential of tissue regeneration. Studies of teleost genetic regulatory elements, such as miRNAs, can provide a much needed insight into the human gene regulatory networks through orthologous gene functional studies. Given the fact that 82% of human genes that are implicated in genetic-related diseases have their orthologs in zebrafish (Howe et al. 2013), many challenges in human medicine can be addressed by better understanding of conserved genes and molecular mechanisms.

The aim of this review is to summarize the recent progress made in teleost miRNA research and discuss areas of future studies. We review the general features of miRNAs, their roles in teleost development and physiology, and give an overview of modeling miRNA functions in teleosts. We discuss the major gaps in knowledge on miRNA in teleosts, particularly in regard to other model systems.

## Overview of miRNA Biology

### Biogenesis and Mechanisms of Action

**Fig. 1.—:**
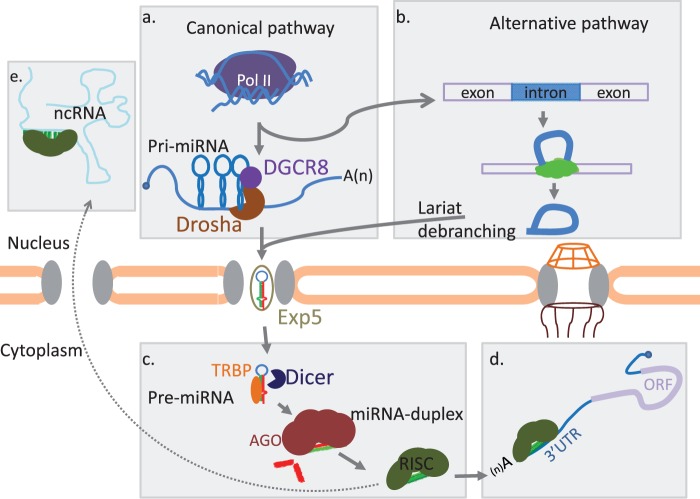
miRNA biogenesis pathways. miRNA processing starts from the nucleus. (*a*) Canonical pathway represents those miRNAs that are transcribed by polymerase II and then are processed by Drosha and associated proteins. (*b*) Alternative miRNA processing pathway represents those miRNAs that bypass Drosha processing; for example, lariats that debranch and form pre-miRNA structure. (*c*) Pre-miRNA processing in cytoplasm by Dicer together with other RNA-binding proteins. (*d*) miRISC binds to 3′-UTR of an mRNA for translational repression. (*e*) miRISC is transported to the nucleus and binds to ncRNAs including pri-miRNA to repress their processing or interfere with their functions.

There are several known pathways of miRNA biogenesis (fig. 1). In the canonical pathway, miRNA synthesis begins in the nucleus, where miRNA genes are transcribed by RNA polymerase II (or polymerase III for some miRNAs) and form capped and polyadenylated primary nascent transcripts (pri-miRNAs) of variable length, ranging from hundreds to thousands ribonucleotides (Cai et al. 2004; Borchert et al. 2006). These transcripts can be monocistronic (single hairpin) or polycistronic (multiple hairpins). A hairpin has three defined features: Terminal loop, internal bulges, and double-stranded stem.

The pri-miRNAs are further processed to shorter (∼70 nt) precursor miRNAs (pre-miRNAs) by Drosha (Lee et al. 2003), an RNase type III enzyme, together with at least 20 other polypeptides, such as DiGeorge syndrome critical region 8 (Dgcr8, also termed as Pasha) and a double-stranded RNA-binding domain protein (Tomari and Zamore 2005) (fig. 1*a*). The resulting hairpin structure has phosphate and hydroxyl groups at its 5′- and 3′-ends, respectively, and a characteristic 2-nucleotide overhang at the 3′-end (Han et al. 2004; Tomari and Zamore 2005; Kim et al. 2009). Pre-miRNAs are regulated by diverse processes (Burroughs et al. 2011). Some miRNAs require additional protein factors, such as p68 or p53 (Fukuda et al. 2007) and coactivator KH splicing regulatory protein (Trabucchi et al. 2009). However, pre-miRNAs can also be formed through Drosha-independent pathway (fig. 1*b*) (Okamura et al. 2007; Ruby et al. 2007). It is not clear, when Drosha-dependent mechanism emerged in metazoan evolution. Nevertheless, mirtrons, that is miRNAs that are processed from mRNA introns by a spliceosome, do exist in distant animal lineages, such as nematodes (Ruby et al. 2007), insects (Okamura et al. 2007), and mammals (Babiarz et al. 2008), as well as in plants (Zhu et al. 2008).

After the transportation of a pre-miRNA from nucleus to cytoplasm, which is facilitated by Exportin-5 in the presence of Ran-GTP (Yi et al. 2003; Bartel 2004; Zeng and Cullen 2004; Kim et al. 2009), pre-miRNA is diced by Dicer, an another RNase III, and associated proteins, such as trans-activator RNA-binding protein, protein activator of PKR (PACT), and Argonaute 2 (Ago2); this process yields in approximately 22-nt-long miRNA duplex (Lee et al. 2002, 2003). However, some miRNAs origin from a Dicer-independent pathway (Cheloufi et al. 2010; Dueck and Meister 2010). In the canonical miRNA processing pathway, one of the strands is loaded to Ago protein through a mechanism unresolved yet (fig. 1*c*). Several hypothetical models explain the incorporation of a single strand to form an active miRNA-induced silencing complex (miRISC), including active incorporation of one of the strands after unwinding by a helicase using ATP (Salzman et al. 2007), ATP-dependent loading of miRNA duplex to Ago but passive process of strand selection (Kawamata et al. 2009) and stepwise loading, wedging, and unwinding by Ago (Kwak and Tomari 2012).

**Fig. 2.—:**
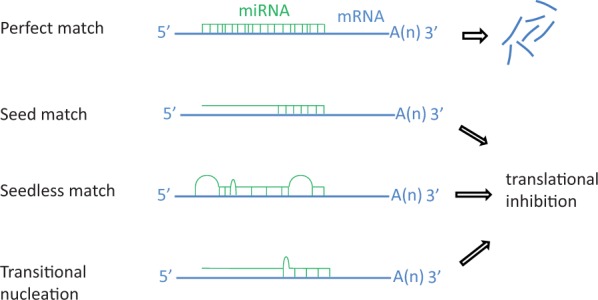
Four miRNA–mRNA interaction models and their final outcome. A perfect pairing between miRNA (green) and mRNA (blue) results in the degradation of mRNA, whereas imperfect matching (only seed, seedless, and transitional nucleation pairings) results in translational inhibition.

The major role of miRNAs in cellular processes is posttranscriptional repression of mRNA in cytoplasm. However, recent studies indicate that mature miRNA can be imported into the nucleus and repress ncRNAs (Leucci et al. 2013). The posttranscriptional suppression is predominantly achieved by binding the miRISC at 3′-UTR of an mRNA. Base complementarity between miRNA and mRNA influences the final outcome of the repression (fig. 1*d*), in which a perfect base pairing results in target degradation, whereas imperfect base pairing yields sequestration of a target (Bartel 2009). The “seed” sequence, nucleotides at the positions 2–8 from the 5′-end of a mature miRNA, is the major determinant of imperfect matching. Seed sequences are highly conserved among species and used to categorize different miRNA families (Griffiths-Jones et al. 2008). Different models have been proposed to explain the mechanism of interaction between miRISC and mRNA, including seed matching, which is the complementarity between the seed and its target mRNA (Lee et al. 1993), seedless matching (Lal et al. 2009), centered paired site (Shin et al. 2010), and pivot pairing and transitional nucleation models (Chi et al. 2012) (fig. 2). In mammals, this interaction can result in reduced translational initiation rate followed by mRNA deadenylation and degradation (Guo et al. 2010; Hu and Coller 2012). Different mechanisms of miRNA-mediated gene silencing have been described (Fabian and Sonenberg 2012).

**Fig. 3.—:**
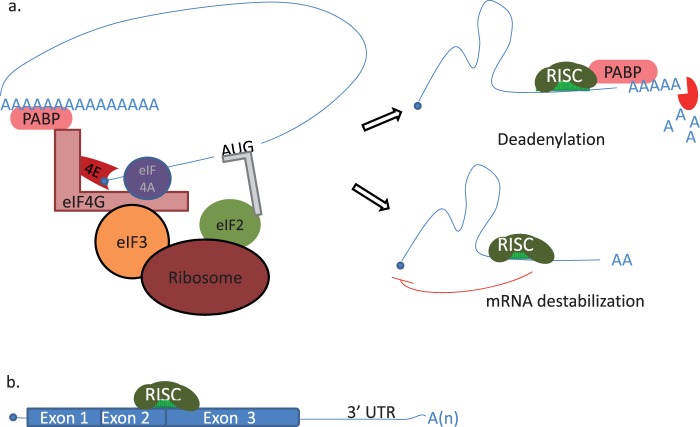
Examples of miRNA mechanisms of action. (*a*) mRNA translation includes initiation, elongation, and termination steps, which are facilitated by the binding of different RNA-binding proteins. Poly(A)-binding protein (PABP) binds to poly(A) tract that in turn binds to eIF4G. eIF4G serves as a platform for the binding of eIF4E (binds to m^7^G cap structure at the 5′-end of mRNA), eIF4A, eIF3, and other proteins. These interactions shape mRNA and enhance the translation (left). However, the interaction of PABP with miRISC augments miRNA-mediated translational repression through the recruitment of deadenylase (right, top). In absence of PABP, miRISC binds to 3′-UTR and destabilizes an mRNA (right, bottom). (*b*) miRISC binds to exons for translational repression possibly by limiting translational elongation. The scheme is simplified and depicts only some of the RNA-binding proteins taking part in the process.

Although the most known function is to mitigate mRNA translation, animal miRNAs are involved also intranslational promotion and repression of other noncoding RNAs. Posttranscriptional repression by miRNAs can be achieved through inhibition of translation initiation, inhibition of translation elongation, premature termination of translation, or deadenylation (Eulalio et al. 2008; Moretti et al. 2012). In zebrafish, translational control by miRISC is largely observed before gastrulation; however, during and after the gastrulation, the action of miRISC is switched to mRNA destabilization (Subtelny et al. 2014). miRISC binds poly(A)-binding protein and recruits deadenylase to promote poly(A)-tail cutting (Fabian et al. 2009) (fig. 3*a*). Pasquinelli and Ruvkun (2002) suggested that miRNAs can bind to the 5′-end of mRNAs, pre-mRNAs, and DNA to suppress translation, facilitate alternative splicing, and form RNA–DNA duplexes, respectively. miRNAs bind not only to untranslated regions (UTRs) but also to exons (fig. 3*b*) (Tay et al. 2008; Fang and Rajewsky 2011). Furthermore, miRNAs can be imported to nucleus and repress other ncRNAs (fig. 1*e*). For instance, MALAT1, a long ncRNA, is a target of miR-9 in the nucleus (Leucci et al. 2013). Moreover, miRNAs have functions in translational promotion; for example, miR-10 is implied in enhancing mRNA translation of a ribosomal protein by binding at the 5′-UTR (Ørom et al. 2008). These different mechanisms highlight the flexibility of miRNA action.

### Regulation

**Fig. 4.—:**
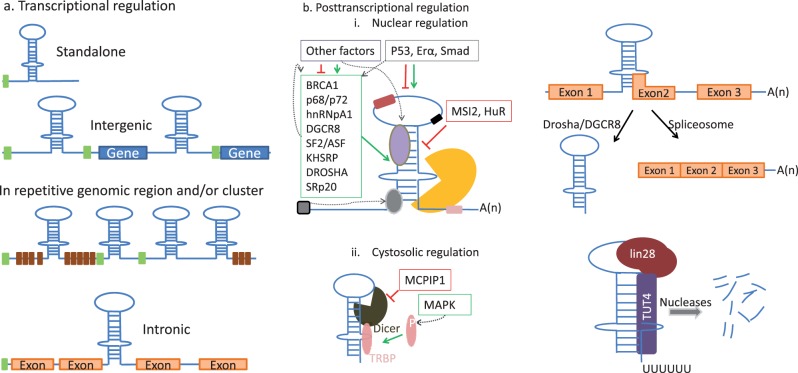
*cis-* and *trans*-acting regulatory elements during miRNA biogenesis (*a*) transcriptional regulation, where the genomic location of a miRNA determines its regulation. Green and brown bars indicate upstream promoter elements and repetitive sequences, respectively. (*b*) Posttranscriptional regulation: (i) Nuclear regulation, where the processing of pri-miRNA to pre-miRNA is determined by direct and indirect interactions with proteins, up- or downstream sequence elements and other factors (left), as well as the competition between microprocessors and spliceosome for a primary transcript that contains a segment of pre-miRNA at its exon–intron junction (right). (ii) Cytosolic regulation, where different factors affect pre-miRNA maturation (left) and degradation (right). See the text for the details. Hammer-headed red lines depict repression, arrow-headed green lines stand for promotion. Gray-dotted lines indicate interaction or alternative pathways.

Strict *cis*- and *trans*-acting regulatory mechanisms exist in a cell to control miRNA biogenesis at different levels. These regulatory steps can be categorized as transcriptional regulation, posttranscriptional nuclear regulation (microprocessor, shuttle, or autoregulation), posttranscriptional cytosolic regulation (processors regulation, loading regulation, or strand selection), and decay (fig. 4).

#### Transcriptional Regulation

miRNA genomic location can be intergenic, intronic, inside repetitive elements, or a standalone gene with its own promoter (fig. 4*a*). Genomic location of a miRNA determines its transcription, which depends on promoter and enhancer elements (Cai et al. 2004; Borchert et al. 2006). A number of miRNAs is clustered. Some miRNA clusters have multiple miRNA promoters inside a cluster, such as C19MC in mammals (Bortolin-Cavaille et al. 2009). Some miRNAs are regulated together with their targets, such as miR-10c and *HoxB4a* in zebrafish (Woltering and Durston 2008). A miRNA and its target can be transcribed as a single transcriptional unit, for example, miR-26b and *ctdsp2* (Han et al. 2012), or miR-412 and *Mirg* (Melamed et al. 2013).

#### Posttranscriptional Nuclear Regulation

The formation of a pre-miRNA involves several factors, including phosphoprotein p53, estrogen receptor alpha (ERα), breast cancer 1 (BRCA1), protein p68, protein p72, splicing factor 2 (SF2/ASF), heterogeneous nuclear ribonucleoproteins, and KH-type splicing regulatory protein (Michlewski et al. 2008; Wu et al. 2010; Suzuki and Miyazono 2011; Kawai and Amano 2012; Sundaram et al. 2013). These factors enhance or inhibit miRNA maturation (fig. 4*a*). For instance, p53 enhances Drosha activity to produce a miR-34 precursor (Tarasov et al. 2007), but it represses the maturation of miR-17-92 cluster (Yan et al. 2009). The binding of KH-type splicing regulatory protein to primary transcripts is essential for the processing of mir-198 (Sundaram et al. 2013). The docking of ERα to Drosha represses pri-miRNA processing of miR-16, miR-145, and miR-195 (Yamagata et al. 2009). Heterogeneous nuclear ribonucleoprotein A1 binds to the loop region of pri-miR-18a to facilitate slicing (Michlewski et al. 2008). Also, tissue-specific inhibition of pri-miR-7 processing by musashi RNA-binding protein 2 (MSI2) and human antigen R (HuR) proteins is reported in mammals (Choudhury et al. 2013). Some of these regulatory proteins act at multiple levels of miRNA biosynthesis pathway (interested readers are referred to Winter et al. 2009; Rinn and Huarte 2011; Suzuki and Miyazono 2011; Finnegan and Pasquinelli 2013). The competition between miRNA processors and spliceosome has been shown in modulating the expression level of mature miRNAs (Ramalingam et al. 2014). Similarly, 52 miRNAs, such as hsa-miR-202, hsa-miR-365, and hsa-miR-412 have active splice sites within a pri-miRNA, which can be a regulatory factor for their expression in tissue- and development-specific manner (Melamed et al. 2013). Also, alternative splicing may uncouple the expression pattern of clustered miRNAs from each other (Ramalingam et al. 2014).

#### Autoregulation

The structure of RNA itself has an important impact on a miRNA biogenesis pathway. miRNAs can regulate own biogenesis through their secondary structure. For example, a miRNA cluster can promote or hinder miRNA processing through accessibility for miRNA processing machineries (Yang et al. 2009). Also, miRNAs are regulated by a feedback loop mechanism with their target (Yang et al. 2009), such as miR-57 and *nob-1*, or miR-7 and SF2/ASF (Wu et al. 2010; Zhao et al. 2010). In other example, let-7 binds at the 3′-end of its primary transcript and enhances its processing (Zisoulis et al. 2012). In addition, Drosha and Dgcr8 regulate each other (Han et al. 2009).

#### Shuttle Regulation

During the pre-miRNA transportation from the nucleus to cytoplasm, Exportin-5 protein protects pre-miRNA from degradation (Winter et al. 2009). The competition between pre-miRNAs and Dicer mRNA for Exportin-5 regulates the mature miRNA homeostasis (Bennasser et al. 2011). Disturbed transport of a pre-miRNA to cytoplasm was observed in several cancer cell lines (Lee et al. 2008; Melo et al. 2010) and during viral infection (Bennasser et al. 2011).

#### Cytosolic Regulation

miRNA maturation process can be further inhibited in cytoplasm (fig. 4*b*); for example, Lin-28 binds to the terminal loop of let-7, and the 3′-end is polyuridylated by terminal uridyl transferases (TUT4/Zcchc11), thereby blocking Dicer processing (Heo et al. 2009). Another RNA-binding protein, MCPIP1, counteracts Dicer processing via cleavage of the terminal loop of a pre-miRNA (Suzuki et al. 2011). Furthermore, phosphorylation of trans-activator RNA-binding protein, which is mediated by MAPK, enhances the stability of the miRNA-generating complex and results in an increase in miRNA production and miRNA-mediated target silencing (Paroo et al. 2009). Loading miRNAs to Ago protein is a critical regulatory step (Suzuki and Miyazono 2011; Treiber et al. 2012), which also determines the target specificity. Some studies showed developmental stage-specific abundance of one of the strands (either guide or passenger one) during the embryonic development of teleosts (Soares et al. 2009; Bizuayehu et al. 2012a). Recent studies show that miRISC can be bound by other transcripts such as circular RNAs (Hansen et al. 2013; Memczak et al. 2013), indicating additional level of regulation of mature miRNAs or different mechanisms of action.

#### miRNA Decay

Half-life of miRNA varies among tissues and miRNA types. It depends on target complementarity, 3′-end modification, cellular condition, and extracellular signaling (Katoh et al. 2009; Rüegger and Großhans 2012). Analysis of miRNA turnover in mammalian embryonic fibroblasts showed the average half-life of miRNA was around 5 days in Dicer1-ablated cells (Gantier et al. 2011), which is by far greater than the half-life of mRNAs, 7.1 h on average (Sharova et al. 2009). However, other research showed faster decay of miRNAs; for example, miR-16 family stability is regulated in a cell cycle-dependent manner, in which the stability increases during the cell cycle exit and decreases during the re-entry (Rissland et al. 2011).

### miRNA Modifications and IsomiRs

miRNA modifications are not stochastic. There are two types of miRNA modifications: Nucleotide modification and nucleotide addition. The former one is an epigenetic mechanism; for example, adenosine (A) deamination results in conversion to inosine (I). A-to-I editing is the most common modification; inosine has similar properties as guanosine (G) in base pairing, thus it can alter the pre-miRNA structure and mature sequences by affecting both miRNA processing and target recognition (Kawahara et al. 2007, 2008). For instance, A-to-I editing inside the seed sequence has been observed in some miRNAs, such as miR-151, miR-376a, miR-376b, and miR-368 (Kawahara et al. 2007; García-López et al. 2013). At least 6% of pri-miRNAs in mammals have A-to-I editing sites. The edited miR-376a was expressed in specific tissues, and it regulated target genes different than the unedited miR-376a (Kawahara et al. 2007). The edited precursors were removed at early postzygotic stages during mouse preimplantation development (García-López et al. 2013).

Apart from A-to-I editing, mature miRNAs can be modified at the 3′-end through uridylation or adenylation (Katoh et al. 2009; Chiang et al. 2010). This type of editing has been found in many miRNAs (Luciano et al. 2004; Kawahara et al. 2007, 2008). It has important implications in miRNA biogenesis and target diversification, because it can affect the secondary structure of pri-miRNAs or miRNA:mRNA base pairing. Thus, it can create differential accumulation of mature miRNAs and target discrimination. The purpose of these modifications is context dependent; for example, adenylation is required for selective stabilization of miR-122 in mouse liver (Katoh et al. 2009), whereas in THP-1 cell line, adenylation reduces effectiveness of miR-26a, miR-27a, and miR-122 (Burroughs et al. 2010). In vitro editing of pri-mir-142 in two positions remarkably reduced pre-mir-142 synthesis (Yang et al. 2006). This indicates that miRNA editing is one of the mechanisms that increase the repertoire of miRNAs and their targets.

Several mature miRNAs have size variants, termed as isomiRs. They are present in divergent species (Lee et al. 2010; Li et al. 2011; Bizuayehu et al. 2012a; Humphreys et al. 2012; Wei et al. 2012; Yi et al. 2013). The origin of isomiRs is still not fully understood. Different mechanisms have been proposed, including degradation or imprecise cleavage of pre-miRNA during processing. However, the occurrence of isomiRs is likely nonrandom. Although the random degradation of mature miRNAs by nucleases cannot be excluded, the differential expression patterns and the observed target differences (Bizuayehu et al. 2012a; Humphreys et al. 2012; Wei et al. 2012) suggest that biosynthesis of isomiRs is a regulated process. This hypothesis is supported by convergent results obtained in divergent species (Fernandez-Valverde et al. 2010; Cloonan et al. 2011; Wei et al. 2012). For example, nontemplate nucleotide additions at 3′-end, mostly A and U, but also C and G, have been reported in zebrafish (Wei et al. 2012), Atlantic halibut (Bizuayehu et al. 2012a), and blunt snout bream (Yi et al. 2013). A and U additions can stabilize or degrade mature miRNAs (Burroughs et al. 2010). Posttranscriptional miRNA modifications, resulting in a nontemplate nucleotide addition, involve a number of enzymes including MTPAP, PAPD4, PAPD5, ZCCHC6, ZCCHC11, and TUT1 (Wyman et al. 2011).

Modifications occur not only at the 3′-end of a mature miRNA but also at its 5′-end. The majority of the 5′-end nucleotide alterations in Atlantic halibut miRNA were isomiRs (Bizuayehu et al. 2012a). It has been suggested that 5′-end size variations could result from the presence of multiple loci with different pre-miRNA structures (Starega-Roslan et al. 2011). In the light of canonical miRNA:mRNA interaction and seed complementarity, the addition or truncation of nucleotides at the 5′-end can alter the target specificity of a miRNA. This has been shown in 5′-isomiRs of miR-101 (Llorens et al. 2013) and miR-133a (Humphreys et al. 2012). However, Cloonan et al. (2011) have shown that both canonical miRNAs and their isomiRs cooperate and have considerable mRNA target overlaps. More studies are needed to uncover whether isomiRs are random degradation or rather regulated biosynthesis products, and whether the action of isomiRs is divergent or redundant.

### miRNA Target Site Polymorphism and Posttranscriptional Modifications

Polymorphism in DNA sequence of the target site influences miRNA-target interaction through stabilizing or destabilizing the existing miRNA target sites, or creating new target sites (Georges et al. 2007). miRNA target site polymorphism can result in phenotypic variation and disease conditions (Saunders et al. 2007; Ziebarth et al. 2012). For instance, a single-nucleotide polymorphism (SNP) at the 3′-UTR of mystotin gene in Texel sheep allows binding by miR-1 and miR-206, which in effect creates muscular hypertrophy (Clop et al. 2006).

In teleosts, target site polymorphism is found in a number of processes, such as muscle development and regeneration, photoreceptor morphogenesis, immune response, or craniofacial development, and it can have functional effects (Loh et al. 2011). In the three-spined stickleback (*Gasterosteus aculeatus*), polymorphic target sites were found at the 3′-UTR of Glyceraldehyde-3-phosphate dehydrogenase, which has two alleles. One allele was predicted as a target of miR-2888, miR-705, and miR-2305, whereas the other allele was targeted by miR-1777b (Chaturvedi et al. 2014). In Lake Malawi cichlids, SNP density in the predicted miRNA target sites was higher than in the flanking regions. Allele frequency analysis and lineage specificity of these sites suggested contribution of miRNA target site polymorphism to species diversification (Loh et al. 2011). Further identification of miRNA target site polymorphism in other teleosts will help to understand the role of miRNA in teleost evolution.

RNA editing has an effect not only on miRNA processing and target diversification; A-to-I and C-to-U editing can also disrupt legitimate miRNA target sites. It has been found that RNA editing sites are highly enriched at “seeds” of miRNA target sites; and this can create new miRNA target positions or disrupt the existing ones (Gu et al. 2012). Editing can alter RNA secondary structure, which can affect the accessibility of miRNA-binding sites (Brodersen and Voinnet 2009).

### Evolutionary Constraints and Teleost Specificity of miRNAs

Selection pressure on mature miRNA is immense. How the potency of miRNA fine tuning of diverse biological pathways is rendered in the course of evolution? This capacity is shaped by various selective forces (internal and external), such as precursor structure, RNA-binding proteins, target-binding sites, target selection, and decoys. The “minimal sufficient” structural requirement of miRNA are as follows: 1) A pre-miRNA must form a stem structure homoduplex, meaning that two segments have to show complementarity with low free energy; 2) an miRNA must fulfill the minimum requirement to be loaded to Argonaute protein, and in the case of animals, no perfect complementarity to Ago catalytic center is required; and 3) miRNA’s seed sequence must have binding site(s) on its target(s). Moreover, miRNA evolution is affected by decoys, such as circular RNAs, long noncoding RNAs, and other small RNAs (Hansen et al. 2013; Memczak et al. 2013). In addition, miRNA can be bound by competing endogenous RNAs (ceRNAs). Therefore, miRNA loci are under both positive and negative selection pressure (Kosik 2013).

Numerous miRNAs are common among divergent animal species (Niwa and Slack 2007). Several lines of evidence indicate that the speciation of metazoans has been accompanied by emerging novel miRNAs (Hertel et al. 2006; Tarver et al. 2013) and that the majority of the inventions have occurred in vertebrates (Heimberg et al. 2008). Teleosts have additional copies of miRNAs as a result of duplicate retention following the teleost-specific whole-genome duplication and gene duplication events. A study on localization of selected pri-miRNA duplicates in zebrafish has been performed (He et al. 2011), but no information is available on the regulation and functional characterization of miRNA duplicates, that is mature miRNA sequences originating from different loci. However, it is possible that cell- or tissue-specific transcriptional factors may induce differential expression of miRNA duplicates. For instance, human miR-365 has two copies, one with an active splice site, whereas the other one is intronic; this suggests regulator modulation of the expression of paralogous miRNAs (Melamed et al. 2013). Previous studies on protein-coding genes demonstrated that several paralogs, which evolved as a result of teleost-specific whole-genome duplication, had distinct pattern of expression and attained subfunctionalization, neofunctionalization, or loss of their function in the course of evolution (Brunet et al. 2006; Crow et al. 2006). Furthermore, some groups of teleosts underwent genome reduction, such as members of orders Tetraodontiformes and Pleuronectiformes (Brainerd et al. 2001; Venkatesh 2003); thus, studying miRNAs in these species in comparison to species that retain duplicates can expand our understanding of the genome duplication event. Functional characterization of miRNA duplicates is important in this context.

## miRNA in Teleost Development

### Early Development

miRNAs are involved in regulation of early developmental transitions. A number of miRNAs has temporally defined expression patterns, such as those expressed during maternal-to-zygotic transition (MZT) and metamorphosis (table 2). MZT is a two-step process of removal of a subset of maternal mRNA and proteins followed by the initiation of zygotic mRNA transcription (Tadros and Lipshitz 2009). It is regulated by the two networks, maternal and zygotic. Maternal mRNA stability, translation, and localization are the three features that determine control of early embryogenesis. miRNA expression profiling experiments have shown the probable role of miRNA in destabilization of maternal transcripts (Wienholds et al. 2005; Tani et al. 2010; Bizuayehu et al. 2012a). mRNA degradation pathway through the miRNA mediation occurs after the MZT (Barckmann and Simonelig 2013). miR-430, which is highly expressed during the blastula stage, is involved in maternal transcript clearance (Giraldez et al. 2006). However, other miRNAs such as miR-34, miR-200a, miR-200b, and miR-206 are also found abundantly during the embryogenesis. For example, miR-206 is expressed both maternally and zygotically in zebrafish, and it is essential in controlling cell movements during the gastrulation (Liu et al. 2012).

**Table 2:** Overview of miRNAs Characterized in Teleosts

Developmental Stage/ Tissue/Process	miRNA	Species	Method	Function	References
Embryonic development	miR-430	Zebrafish	Cloning, knockdown, qRT-PCR	Maternal transcript clearance	Giraldez et al. (2006)
Gonadal development	miR-430	Zebrafish	Knockdown, miRNA target protection assays, reporter assays, transgenics, ISH, qRT-PCR	PGC migration	Staton et al. (2011)
Metamorphosis	let-7	Japanese flounder Atlantic halibut	NGS and qRT–PCR	Larval to juvenile transformation	Bizuayehu et al. (2012a) and Fu et al. (2013)
Vascular development	miR-126	Zebrafish	Microarray, knockdown, luciferase reporter assay, qRT-PCR	Angiogenesis	Fish et al. (2008)
Vascular development	miR-142-3p	Zebrafish	Knockdown, overexpression, qRT-PCR	Vascular integrity, remodeling and angiogenesis	Lalwani et al. (2012)
Vascular development	miR-221	Zebrafish	NGS, ISH, northern blot, qRT-PCR, knockdown, miRNA sensor assays	Endothelial tip cell proliferation and migration	Nicoli et al. (2012)
Vascular development	let-7g, miR-23b, miR-27a, miR-27b, miR-29a, and miR-126	Zebrafish	microarray, northern blot, ISH, qRT-PCR, knockdown, luciferase reporter assay	Arterial-venous segregation, angiogenesis, branching and tip cell specification	Biyashev et al. (2012)
Oocyte and early embryo	miR-21, miR-23a, miR-26a, miR-30d, miR-92a, miR-125a, miR-125b, miR-126-5p, miR-126-3p, miR-200b, and miR-455	Rainbow trout	Cloning and qRT–PCR	?	Ramachandra et al. (2008)
Oocyte and early embryo	miR-34	Zebrafish	Knockdown, microarray, qRT–PCR	Nervous system development	Soni et al. (2013)
Oocyte	miR-15, miR-29, miR-92, miR-101, miR-126, miR-181-3p, miR-196, miR-202-5p, miR-202-3p, miR-221, miR-301, miR-338, and miR-2184	Rainbow trout	Microarray	?	Juanchich et al. (2013)
	let-7, miR-10, miR-21, miR-24, miR-25, miR-30, miR-143, miR-146, miR-148, and miR-202	Rainbow trout	NGS	?	Ma et al. (2012)
Brain	let-7g, k, h, i, l, miR-29a, b, miR-103, miR-124a, b, c, d, and miR-125	Asian seabass	qRT–PCR	?	Xia et al. (2011)
	let-7a, b, c, and d, miR-9, miR-21, miR-124, miR-135c	Zebrafish	NGS, qRT–PCR	?	Soares et al. (2009)
	let-7a,b,c,f,i, miR-7b, miR-9-5p, miR-9-3p, miR-34b, miR-103, miR-107, miR-124a, miR-125a,b, miR-128, miR-129-3p, miR-132, miR-138, miR-181a,b, miR-216, miR-217, miR-219, and miR-375	Zebrafish	Microarray, ISH	?	Wienholds et al. (2005)
	let-7a,b,c, miR-9, miR-34, miR-92b, miR-124, miR-128, miR-135c, miR-137,miR-138, miR-153a, miR-219, miR-222	Zebrafish	ISH	?	Kapsimali et al. (2007)
	miR-7 and miR-9	Zebrafish	Gain- and loss-of-function	Brain boundary organization	Leucht et al. (2008) and Memczak et al. (2013)
Eye	miR-124	Zebrafish	NGS, qRT–PCR	?	Soares et al. (2009)
	let-7b, miR-9, miR-30a, miR-92b,miR-96 miR-124, miR-181a,b, miR-182, miR-183, miR-184, and mir-204	Zebrafish	ISH	?	Kapsimali et al. (2007)
	miR-204	Medaka	Knockdown, ISH, luciferase reporter assays, qRT-PCR	Lens development	Conte et al. (2010)
	let-7	Zebrafish	Luciferase reporter assays, qRT-PCR, knockdown	Müller glia cells differentiation	Ramachandran et al. (2010)
	miR-7 and miR-454a	Zebrafish and medaka	ISH	?	Ason et al. (2006)
	miR-7, miR-9, miR-34b, miR-96, miR-124a, miR-125b, miR-132, miR-181b, miR-182, miR-183, miR-184, and miR-204, miR-215, miR-216, miR-217	Zebrafish	Microarray, ISH	?	Wienholds et al. (2005)
	let-7g,n,k,h,i,l, miR-21c, miR-29a,b, miR-124, miR-125, miR-126a,b, miR-181a,b, miR-183a,b, miR-184a,b	Asian seabass	qRT–PCR	?	Xia et al. (2011)
Heart	miR-218a-1/2	Zebrafish	Knockdown, overexpression, ISH, luciferase reporter assay, qRT-PCR	Heart field migration	Fish et al. (2011)
	miR-138	Zebrafish	Knockdown, antagomiR, ISH, luciferase reporter assay, qRT-PCR	Cardiac patterning	Morton et al. (2008)
	miR-21, miR-218a	Zebrafish	Knockdown, overexpression, ISH, qRT-PCR, luciferase reporter assay	Heart valve formation	Chiavacci et al. (2012) and Banjo et al. (2013)
	let-7e,f,g,h,i,j,k,l,m,n,o, miR-1a, miR-20, miR-21a,b,c, miR-29a,b, miR-103, miR-125, miR-126a,b, miR-128c, miR-145, and miR-199b	Asian seabass	qRT–PCR	?	Xia et al. (2011)
	miR-1, miR-101a, miR-130b,c, miR-133a, miR-221, and miR-499	Zebrafish	NGS, qRT–PCR	?	Soares et al. (2009)
	let-7i, miR-15b, miR-17a-3p, miR-21, miR-92b, miR-128, miR-133, miR-146a,b, miR-150, miR-194a, miR-204, miR-210-3p, miR-301a, miR-429, miR-730, miR-733, miR-738,	Zebrafish	Microarray, northern blot, qRT-PCR, ISH	Regeneration	Yin, Lepilina, et al. (2012)
Muscle	miR-1, miR-21, miR-133a,b,c, miR-203b	Zebrafish	NGS, qRT–PCR	?	Soares et al. (2009)
	miR-203b	Nile tilapia	qRT–PCR and luciferase reporter assay	Muscle development	Yan, Guo, et al. (2013)
	let-7a,c,f, miR-1, miR-17a, miR20a,b, miR-126, miR-133c, miR-181a, miR-203b, miR-206, miR-214, and miR-738	Zebrafish	Microarray, northern blotting, qRT–PCR	Transition from hyperplasia to hypotrophy	Johnston et al. (2009)
	miR-499	Zebrafish	ISH, overexpression, knockdown, transgenics	Muscle fiber-type specification	Wang et al. (2011)
	miR-140	Zebrafish, medaka	Knockdown, reporter assays, cloning, ISH	Palatogenesis	Ason et al. (2006) and Eberhart et al. (2008)
	let-7b,c,j, miR-1, miR-15a, miR-22a, miR-27a, miR-30b, miR-34, miR-125b, miR-133a,miR-140, miR-152, miR-192, miR-193a, miR-199, miR-204, miR-206, miR-214, miR-218a,b, miR-301c, and miR-460	Nile tilapia	NGS, qRT-PCR, microarray	Growth	Huang et al. (2012) and Yan et al. (2012a)
	miR-1, miR-21, miR-23a, miR-24, miR-26a, miR-27a,b, miR-29b, miR-125b, miR-133a-3p, miR-155, miR-181a-5p, miR-206, miR-214, miR-221, and miR-222	Common carp	NGS, qRT-PCR	?	Yan et al. (2012)
	miR-1, miR-9b-3p, miR-10b, miR-10d-5p, miR-23b, miR-92a, miR-122, miR-133b-5p, miR-135a,c, miR-144-3p, miR-144-5p, miR-145, miR-193b, miR-212, miR-462, miR-551, and miR-2187-5p	Blunt snout bream	NGS	Growth	Yi et al. (2013)
	miR-1 and miR-133	Zebrafish	Transgenics, microarray, luciferase reporter assay, knockdown, qRT–PCR	Actin organization in the sarcomere and its function	Mishima et al. (2009)
Fin	mi-R2a, miR-26a, miR-66, miR-69, miR-80, miR-144, miR-200b, miR-203, miR-301, and miR-338	Zebrafish	Microarrays, reporter assays, knockdown	Regeneration	Thatcher et al. (2008)
	miR-7a, miR-17-5p, miR-20a, miR-21, miR-22a, miR-23, miR-25, miR-26a, miR-31, miR-92b, miR-100, miR-101, miR-124, miR-133,miR-137, miR-138, miR-146, miR-182, miR-187, miR-194b, miR-196a, miR-200a,c, miR-205, miR-216a, miR-301, miR-338,	Zebrafish	Microarray, northern blot, qRT-PCR, ribonuclease protection assays	Regeneration	Yin et al. (2008)
Ionocytes, nasal epithelium, neuromasts, pronephros, and scattered epithelial cells	miR-8, miR-141, miR-200a, miR-200b, miR-200c, and miR-429	Zebrafish	ISH, northern blot, reporter assays	Osmoregulation	Flynt et al. (2009)
Kidney and gills	miR-30c and miR-429	Nile tilapia	qRT-PCR, luciferase reporter assay	Osmoregulation	Yan, Zhao, et al. (2012) and Yan et al. (2012b)

Note.—?, function unknown; ISH, *in situ* hybridization; NGS, next-generation sequencing; qRT-PCR, quantitative real-time polymerase chain reaction.

Several studies demonstrated the role of miRNAs in the progression of teleost embryonic development. Zebrafish embryos and larvae lacking zygotic Dicer1 had slow growth rate and survived only for 2 weeks (Wienholds et al. 2003). Similarly, maternal and zygotic Dicer mutant zebrafish embryos had morphogenetic defects during the gastrulation, brain formation, somitogenesis, and heart development (Giraldez et al. 2005). In the latter study, injections of mature miR-430 into the Dicer-deficient embryos partially rescued the gastrulation and reduced brain ventricle morphogenesis defects, indicating its role in the processes. In addition, miR-430 is transcribed after the zygotic genome activation by maternally stocked transcriptional factors, such as Nanog, Pou5f1, and SoxB1 (Lee et al. 2013), indicating zygotic origin of this miRNA. However, miR-34 is a maternal miRNA involved in early neural system development (Soni et al. 2013). The roles of other maternally stocked miRNAs, such as miR-24, miR-30, miR-126, miR-146, and miR-221 (Ma et al. 2012; Juanchich et al. 2013) remain to be uncovered.

### Organogenesis

Several species of miRNAs have been characterized during teleost organogenesis (table 2).

As a part of the transcript pool, miRNAs create a context for the organ to be formed. Rudiment formation of any organ needs organized causality, which starts with signaling followed by consequential changes in transcripts pool management. These sequential actions require superseding the transcripts, buffering the noise from unintended transcripts, and shaping the transcriptional output to fit the context or keep homeostasis. miRNAs help to establish discrete domains of gene expression during organogenesis. Below we discuss known or anticipated roles of some miRNAs in the formation of different teleost tissues.

#### Brain Formation

Diverse types of miRNAs are present in distinct regions of brain (Kapsimali et al. 2007), implying constricted function in a given region. In teleosts, conserved brain-specific miRNAs are found in divergent species (Soares et al. 2009; Xia et al. 2011; Bizuayehu et al. 2012b; Zhu et al. 2012; Xu et al. 2013). Zebrafish maternal and zygotic Dicer mutant embryos showed proper developmental progression of neural plate to neural rod; however, a considerable impairment in neural development was observed in the formation of the neurocoel and neural tube, as well as reduction of the brain ventricles and lack of distinct brain regions were found. These defects were partially rescued by injection of a preprocessed miR-430 family to the mutant (Giraldez et al. 2005).

The expression of brain miRNAs depends on the cell status; for example, miR-92b is widely expressed in proliferative neural cells regardless of the fate of these cells, whereas miR-124 is expressed in differentiated neurons only. In contrast, miR-9 and miR-135c are expressed in both cell types (Kapsimali et al. 2007). In that study, the authors also showed that miR-181a and b were expressed specifically in retina cells.

miRNAs have brain-organizing activity; for instance, miR-9 is expressed selectively in late embryonic neural tube by sparing the midhind brain to define the boundary (Leucht et al. 2008). Other study showed that loss of miR-7 could result in specific reduction of midbrain size without affecting the telencephalon at the anterior tip of the brain (Memczak et al. 2013). In summary, localized, transient, and constitutive expression of miRNAs in teleost brain indicates their function in brain morphogenesis and maintenance of distinct subregions and cell types.

#### Eye Formation

The embryonic origin of teleost eye is similar to other vertebrates; however, vision in teleost depends on ecological niche and behavior of a species. The vision procures adaptation to the environment, thus eye tissue-specific gene expression guides this adaptation. Eye development has been well characterized in zebrafish using morphology, gene expression, and in situ labeling (Fadool and Dowling 2008; Gestri et al. 2012). During gastrulation, the middle part of anterior neural plate is destined to be an eye field. This field is under the control of different signaling pathways, which influence the development of the eye. Among them, Wnt signaling pathway defines regions of anterior neural plate including eye field and migration of eye field cells, and promotes eye formation (Cavodeassi et al. 2005). The induction of the eye field is followed subsequentially by a formation of optic cup through invagination of optical vesicles, change of the optic stalk to optic nerve and retina, and by the closure of choroid fissure (Gestri et al. 2012). This remodeling is regulated by many transcriptional factors, such as Meis2, Mitf, Pax2, Pax6, Six3a, Vax1, and Vax2, and signaling pathways, such as Fgf, Hh, Shh, and Wnt (Macdonald et al. 1997; Chow and Lang 2001; Cavodeassi et al. 2005; Conte et al. 2010).

Several miRNAs, such as miR-96, miR-124a, miR-181a, miR-181b, miR-182, miR-183, miR-184, and miR-204 are expressed in eye of zebrafish embryo (Cavodeassi et al. 2005). Similarly, a number of miRNA species was identified in Asian seabass (*Lates calcarifer*) eye (Xia et al. 2011). Spatial localization of miRNAs revealed cell type- and developmental stage-specific expression patterns (Kapsimali et al. 2007). For instance, miR-181a and b were expressed specifically in retina cells (Kapsimali et al. 2007). The authors also showed restricted expression of miR-92b and let-7b in the ciliary marginal zone of the retina and complete absence of these miRNAs in mature retinal neurons. miR-9 was expressed in mature amacrine cells of the inner nuclear layer and in maturing cells of ciliary marginal zone of the retina. miR-30a, miR-184, and mir-204 were localized in lens. Conte et al. (2010) have showed that miR-204 targets *meis2* and modulates Pax6 transcriptional pathway in medaka *Oryzias latipes*. Using morpholino-based knockdown approach, they demonstrated that the depletion of miR-204 resulted in a number of eye development malformations including eye cup impairment, small eyed embryos, impaired lens development, defect in lens epithelial cells patterning, misplacement and disorganization of primary fiber cells, lens herniation, and failure of optic fissure closure. The evolutionary conservation of Pax6-miR-204 pathway is demonstrated in mouse ocular tissues (Shaham et al. 2013). Other miRNAs are found in keeping homeostasis of cells of the eye; for example, let-7 maintains Müller glia cells in a differentiated state (Ramachandran et al. 2010).

Given the need for the precise and intricate regulation of gene expression during eye development and the adaptive significance of eye, further work on developmental and physiological functions of miRNAs in the eye of various teleosts from different ecological niches would disclose the adaptive role of miRNAs in teleosts by using visual system as a model.

#### Muscle Formation

Muscle formation in teleosts begins during the embryonic development with the formation of precursor myogenic cells. These myogenic cells differentiate into a myotome, which has four cell lineages: Muscle pioneers, slow muscle, fast muscle, and medial fast muscle (Johnston 2006). Specification of these lineages is regulated by myogenic regulatory factors (MRFs). MyoD, a member of MRF, controls several downstream genes involved in myogenesis. MyoD is directly regulated by miR-203b in Nile tilapia (*Oreochromis niloticus*) (Yan, Guo, et al. 2013). In zebrafish, myotube production ceases at 40% of the total body length and the transition from hyperplasia to hypotrophy is facilitated by miRNAs, including let-7, miR-19, and miR-130 families (Johnston et al. 2009).

During skeletal myogenesis, myoblasts differentiate into slow-twitch or fast-twitch muscle fibers. These lineage-specific pathways are established by the activity of either slow-twitch restricted or fast-twitch restricted genes, as well as transcription factors (Chauvigne et al. 2005; Elworthy et al. 2008). Lineage-restricted expression of miR-499 leads to the establishment and maintenance of slow-twitch muscle fibers through repression of Sox6, which promotes fast-twitch muscle differentiation; this mechanism is conserved among vertebrates (Wang et al. 2011). Several miRNAs have been identified in skeletal muscle of common carp, and some of them are unique for teleosts (Yan et al. 2012). In zebrafish embryos, miR-1 and miR-133 were implicated in shaping sarcomeric actin organization (Mishima et al. 2009). Further exploration of the function of these unique miRNAs is important to understand muscle formation in teleosts. The role of miRNA in muscle growth is addressed in another section.

#### Cardiovascular Formation

At the early stage of teleost organogenesis, beating heart is a recognized developmental stage because of its visibility ahead of other discernible organs. The heart differentiates from two distinct cardiac progenitor cells termed the first heart field and the second heart field (De Pater et al. 2009; Grimes et al. 2010). miRNAs participate in the regulation of migration of the heart fields to the midline; for example, miR-218a-1/2 titrates roundabout homolog 1 (*robo1*) to regulate endocardial migration through vascular endothelial growth factor (Vegf) signaling (Fish et al. 2011). In zebrafish, the first heart field gives rise to heart tube and progenitors of the second heart field differentiate to form smooth muscle and myocardium (Hami et al. 2011). The heart tube loops, tightens, and forms atrium and ventricle, two chambers delineated morphologically, molecularly, and functionally. miR-138 knockdown during zebrafish cardiac development resulted in defects in elongation of ventricular cardiomyocytes and in early cardiac looping (Morton et al. 2008). In the further analysis, the authors showed that miR-138 had a restricted expression in atrioventricular canal, and it targeted atrioventricular canal domain genes by regulating retinoic acid synthesis and direct repression of chondroitin sulfate proteoglycan 2 (*cspg2*). In zebrafish during heart development, miR-218a interacts with transcriptional factor Tbx5 (Chiavacci et al. 2012), which is necessary for endocardial cell differentiation and valve tissue formation (Camarata et al. 2010). Also, miR-21 is crucial in regulation of heart valve formation by modulating the expression of sprout homolog (*sprout*), programmed cell death 4 (*pdcd4K*), and phosphatase and tensin homolog B (*ptenb*) (Banjo et al. 2013). miR-126 knockdown resulted in collapsing lumens and compromised endothelial tube organization by repressing sprouty-related EVH1 domain-containing protein 1 (*spred1*) and phosphoinositide-3-kinase, regulatory subunit 2 (*pik3R2*) to promote Vegf signaling during zebrafish vascular development (Fish et al. 2008). Similarly, Lalwani et al. (2012) using knockdown and overexpression assays showed that miR-142-3p targeted *cdh5* and influenced vascular integrity, remodeling, and angiogenesis. Also, let-7 family, miR-20b, miR-31, miR-221, and miR-181a promote angiogenesis and lymphangiogenesis in zebrafish (Biyashev et al. 2012; Nicoli et al. 2012; Dunworth et al. 2013). Studies in mammals documented several interesting functions of miRNAs during cardiac morphogenesis; similar studies in teleosts would be valuable to determine miRNA functional conservation in heart development.

#### Gametogenesis

Primordial germ cells (PGCs) are the carrier of the genetic information from one generation to another; therefore, molecular events during their developmental progression must be strictly regulated to ensure a stable transmission of genetic information to future generations. In model fishes, gametogenesis starts from asymmetric mitotic divisions of PGCs, which are specified very early during embryogenesis. PGCs migrate to the future genital ridges and become gonocytes, then during sex differentiation, they transform to spermatogonia or oogonia (Lubzens et al. 2010; Schulz et al. 2010).

PGC specification necessitates the suppression of somatic lineage programs. Depending on the species, PGCs are transcriptionally inert at the beginning (Nakamura and Seydoux 2008; Venkatarama et al. 2010). PGCs have differential stockpile of proteins and transcripts compared with that of somatic cells. One of the mechanisms securing selective mRNA transcript profile in PGCs has been discovered in zebrafish (Mishima et al. 2006). miR-430, a major “clearance” miRNA during the early embryonic development, suppresses some transcripts, such as *nanos*, *tdrd7,* and *hub* in somatic cells but not in PGCs (Mishima et al. 2006; Kedde et al. 2007; Mickoleit et al. 2011). *Nanos*, *tdrd7,* and *hub* are necessary for proper migration, maintenance, and survival of PGCs (Köprunner et al. 2001; Mickoleit et al. 2011), and Dead end (Dnd), an RNA-binding protein, protects 3′-UTR-binding sites of these transcripts from miR-430-mediated repression in PGCs (Kedde et al. 2007). Morpholino-mediated knockdown of *dnd* in several teleost species leads to removal of protection of key PGC-specific transcripts from miR-430-guided suppression, and consequently PGC development is arrested, germline lineage is lost, and the developing individuals are irreversibly sterile (Weidinger et al. 2003; Fujimoto et al. 2010).

After specification in an early embryo, PGCs migrate to the future gonadal ridge. This migration is guided by a chemokine Sdf-1 signaling from the neighboring somatic cells recognized by CXCR4 receptor (Doitsidou et al. 2002; Knaut et al. 2003). This mechanism, found for the first time in zebrafish (Doitsidou et al. 2002), is well conserved across the investigated vertebrates (Stebler et al. 2004). In this migratory route, the role of miR-430 has been demonstrated in the clearance of *sdf-1a* mRNA from previous expressing domains to ensure correct migration of PGCs (Staton et al. 2011).

miRNAs are essential for proliferation and maintenance of germ cell-supporting somatic cells, such as Sertoli and Leydig cells in the testis, and follicle cells in the ovary. Sertoli cell number is a limiting factor in sperm production in a sexually mature fish (Schulz et al. 2010). In Sertoli cell-specific Dicer conditional knockout mouse model, miR-125a-3p, miR-872, and miR-24 have role in translational control during spermatogenesis (Papaioannou et al. 2011). Rakoczy et al. (2013) reported up to 2.6-fold increase in the number of Leydig cells in miR-140-3p ablated mice. Also, miR-202-5p/3p transcripts were identified as potential regulators of mouse embryonic gonad differentiation with strong expression in Sertoli cells (Wainwright et al. 2013). Multiple miRNAs are implicated in granulosa cells apoptosis, steroidogenesis, and cell proliferation (Donadeu et al. 2012; Hu et al. 2012; Yang et al. 2012; Yin et al. 2012; Zhang et al. 2013b). Functional involvement of miR-224, miR-378, and miR-382 in regulation of aromatase expression during follicle development has been demonstrated, and miR-21 promoted follicular cell survival during the ovulation (Donadeu et al. 2012). No such data exist in fish despite the fact that in seasonally reproducing teleost species, proliferation and apoptotic processes in gonads are orchestrated by a complex regulatory network (Nagahama 1994; Chaves-Pozo et al. 2005; Almeida et al. 2008).

#### Skeletogenesis

Skeletal formation includes cartilaginous state (chondrification) and ossified state (ossification) and is regulated by several ubiquitous and specific genes. The major part of skeleton originates from the neural crest, lateral plate mesoderm, paraxial mesoderm, and notochord. Mesenchymal cells become chondrocytes, osteoblasts, or other skeletal cells depending on transcriptional factors and signaling pathways (Karsenty and Wagner 2002). Transcriptional factor *sox9*, for example, is essential for morphogenesis of condensation and cartilage differentiation in zebrafish (Yan et al. 2002) and regulates the expression of miR-140 (Nakamura et al. 2012). However, miR-140 acts independently of *sox9* in the regulation of palatal skeleton development by modulating pdgf-receptor alpha, which is required for migration of palatal precursor and neural crest cells (Eberhart et al. 2008). Similarly, Runx2 is a transcriptional factor that regulates osteoblast differentiation (Flores et al. 2006). Huang et al. (2010) showed direct negative regulation of *Runx2* by miR-204/miR-211 in stroma and myoblast cell lines. These results indicate the importance of miRNAs during bone and cartilage formation but still those few miRNAs that are profiled as skeleton specific are not characterized functionally, particularly in the context of the prevailing problem of skeletal deformities experienced in fish production (Silverstone and Hammell 2002; Bardon et al. 2009).

## miRNA in Growth and Regeneration of Teleosts

### Growth

Muscle cell-type specification requires Hedgehog, fibroblast growth factor, and retinoic acid signaling pathways, as well as T-box genes (Lewis et al. 1999; Ochi and Westerfield 2007). Both coordinated spatiotemporal action of regulatory factors and the removal of certain domains from the previously expressing cells are essential in the cell specification. In zebrafish, miR-214 enhances the cellular response to Hedgehog signaling and facilitates strict specification of muscle cell types through the repression of *su*(*fu*), which is a negative regulator of Hedgehog signaling (Flynt et al. 2007). Bone morphogenetic protein and transforming growth factor beta (TGFβ) signaling pathways modulate the activity of some proteins, such as Smad1, p68, Drosha, or Dgcr8, which are involved in miRNA biogenesis (Suzuki and Miyazono 2011). Once the cell commitment occurs, differential regulation of various factors promotes the differentiation and proliferation, which allow subsequent tissue development.

Fish growth is modulated by spatiotemporal expression of various genes. In rainbow trout (*Oncorhynchus mykiss*) alevins, the transition from endogenous (yolk sac stage) to exogenous feeding changes the expression of metabolic genes and miRNAs (Mennigen et al. 2013), indicating the participation of miRNAs in metabolic pathways of teleost fish during their early growth. Huang et al. (2011) have reported a negative feedback circuit in which insulin-like growth factor 1 (IGF-1) promotes miR-133 expression, which, in turn, represses IGF-1 receptor (IGF-1R) affecting skeletal myogenesis. In Nile tilapia (*O. niloticus*), miR-206 targets *IGF-1* and inhibits its action (Yan, Zhu, et al. 2013), indicating the importance of miRNAs in hypothalamic–pituitary pathway.

Growth of skeletal muscle mass in fish occurs through hypertrophy and hyperplasia of muscle fibers, which absorb myoblasts differentiated from myogenic precursor cells (Johnston 2006). Transcriptional factors, signaling proteins, and ncRNAs are the determinants of muscle mass formation from the first cell commitment to the last fusion stages. These steps include myogenic progenitor cells specification, activation, proliferation, cell cycle exit, differentiation, migration, and fusion. In early embryogenesis of teleosts, specifically during gastrulation, the commitment of somatic cells to be myogenic cells is ignited by MRFs, which also stimulate muscle-specific miRNA biogenesis (Sweetman et al. 2008). On the other hand, miRNAs regulate the level of MRFs. In zebrafish, Goljanek-Whysall et al. (2011) demonstrated elevated expression of *pax3* in dermomyotomal progenitors, but its downregulation in the MRF-expressing myotome. In their model, MRFs activate miR-1/miR-206 in the committed myoblasts, and these miRNAs target residual *pax3* during the progenitor-to-myoblast transition, and they control transitional timing by repressing *pax3*.

Several miRNAs regulate teleost skeletal muscle growth. For example, Huang et al. (2012) reported significant differential expression of skeletal muscle miRNAs between fast-growing and slow-growing strains of Nile tilapia, indicating possible application of miRNAs as selection markers for aquaculture industry. In another study, four miRNAs (miR-1, miR-27a, miR-133a, and miR-206) were differentially expressed during skeletal muscle development of Nile tilapia (Yan et al. 2012a). Similarly, by comparing skeletal muscle of different stages (larvae, 1-, and 2-year old) of common carp (*Cyprinus carpio*), Yan et al. (2012) reported an increase in miR-1, miR-21, miR-133a-3p, and miR-206 expression with age. Earlier in situ hybridization study in zebrafish showed localization of expression of these miRNAs in skeletal muscle (Wienholds et al. 2005). In adult zebrafish, Johnston et al. (2009) demonstrated significant differences in miRNA expression between two fast muscle phenotypes, myotube recruiting and ceased. Further investigation of the relation between miRNAs and MRFs is essential in understanding skeletal muscle growth.

Growth depends on feed intake and is influenced by a number of intrinsic and extrinsic factors (Hoskins and Volkoff 2012), which modulate the expression of miRNAs and their targets. For example, in rainbow trout alevins, a change from endogenous to exogenous feed is associated with the high expression of miR-143 and inverse expression of its target *abhd5* (Mennigen et al. 2013), a gene that activates adipose triglyceride lipase. Feed intake depends on appetite, which is regulated by hypothalamus. Several appetite stimulators/orexigenes (e.g., orexins, neuropeptide Y, and ghrelin) and inhibitors/anorexigenes (e.g., cholecystokinin, leptin, and amylin) have been isolated in fish (Volkoff et al. 2005). The roles of miRNAs in orexigenic and anorexigenic pathways would be the important area of investigation to utilize miRNAs as markers for selective breeding programs.

Sexual dimorphism in growth is observed in many teleost. This difference is largely associated with reproduction-related features, such as timing of maturation, territorialism, ornamentalism, courtship, nest guarding, and nursing, in which the amount of spent energy affects the growth of one of the sexes particularly (Hendry and Berg 1999). Various studies have shown the difference in expression of genes implicated in sexual growth dimorphism; however, few studies have been performed on sexually dimorphic expression of miRNAs in fish tissues (Bizuayehu et al. 2012b) not directly related to the growth of fish. Scientific knowledge of the genetic basis of growth traits provides an avenue for improvement in aquaculture, and miRNAs can be assistive in such the endeavor.

miRNAs are implicated in nutrient metabolism. Fasting and re-feeding experiment in rainbow trout showed significant upregulation of miR-122 and miR-33 together with *cpt1a* and *cpt1b* in liver, suggesting lipogenic role of miRNAs at multiple levels of the hepatic intermediary metabolism (Mennigen et al. 2012). Twenty-seven growth-related miRNAs were identified in blunt snout bream *Megalobrama amblycephala*, including miR-23b, miR-92, and miR-462, which were expressed abundantly in slow-growing groups compared with fast-growing groups (Yi et al. 2013). The predicted targets of these miRNAs were involved in metabolic pathways.

### Regeneration

Teleosts grow continuously throughout their life, and they are able to regenerate organs and appendages, including spinal cord, heart, retina, scales, and fins. Although similar molecular and cellular processes exist in both mammals and fish, regenerative capacity of adult mammals is limited when compared with fish. Concentration or spatiotemporal availability of developmental regulators might constitute a difference in regeneration capability between mammals and teleosts (Kawakami et al. 2006). A number of studies have been conducted to elucidate biological mechanisms that govern regeneration, but still there are unidentified signals that initiate and regulate certain regenerative steps. Epimorphic regeneration requires signaling pathways, such as Wnt, fibroblast growth factor, retinoic acid and Hedgehog, or notch (Poss et al. 2003; Kawakami et al. 2006; Blum and Begemann 2012; Weidinger et al. 2013). The requirements for a signal can be similar or variable among the regenerative tissues (Duszynski et al. 2013). As modulators of transitional events, miRNAs participate in the regulation of regeneration process through temporal clearance of unneeded transcripts.

Well-investigated regeneration of caudal fin in teleosts has three main discernible stages: 1) wound healing, closing of wound by the migration of epithelial cells; 2) blastema formation, disorganization of mesenchymal tissue followed by formation of a mass of undifferentiated, proliferating mesenchymal progenitor-like cells; and 3) regenerative out-growth, proliferation, and differentiation of blastemal cells (Poss et al. 2003). Numerous miRNAs are down- or upregulated during the fin regeneration. For instance, miRNA microarray experiment showed the upregulation or maintenance of 6 miRNAs and downregulation of 16 miRNAs by Fgf signaling (Yin et al. 2008). Massive differences in miRNA expression are reported between intact, amputated, and regenerating fins of zebrafish (Thatcher et al. 2008). This report indicated that *bmp3*, *hsp60,* and *msxb* genes had role in regeneration and were predicted targets of up- or downregulated miR-200b, miR-2/miR-338, and miR-301, respectively. In gain- and loss-of-function experiments, Yin et al. (2008) showed that the regulated depletion of miR-133 resulted in effective fin regeneration. Similarly, miR-203, which has binding sites on the 3′-UTR of transcriptional factor *lef1*, has been significantly downregulated during fin regeneration (Thatcher et al. 2008). *Lef1* is involved in blastema formation and marks the basal epidermal layer and distal blastema (Poss et al. 2000). miR-203 represses *left1* and in consequence blocks the fin regeneration (Thatcher et al. 2008). In contrast, loss of miR-203 results in abundance of *lef1* and fin overgrowth, indicating the importance of miR-203 not only in blastema formation but also for proper termination of the regeneration process (Thatcher et al. 2008). These findings indicate the reprogramming of miRNA expression in a tissue in response to the regeneration program.

Cardiac tissue regeneration in zebrafish occurs through cardiomyocyte dedifferentiation, transdifferentiation, and proliferation (Jopling et al. 2010; Zhang et al. 2013c). Alteration of expression of several miRNAs during cardiac regeneration (upregulation of 10 and downregulation of 8 miRNAs) was observed at 7 days postamputation (Yin, Lepilina, et al. 2012). Further evaluation of miR-133 in this study indicated that miR-133 had several targets, among them *mps1* and *cx43*, which are essential for the regeneration process. Given that miR-133 has regulatory role in skeletal muscle proliferation (Chen et al. 2006), the repression of miR-133 during heart regeneration may indicate reprogramming.

miRNAs are implicated in neuronal regeneration, including central nervous system and retina. Repressive action of miR-133b toward *rhoA* mRNA, which inhibits axon regrowth, has been demonstrated during spinal cord regeneration (Yu et al. 2011). In addition, the authors have demonstrated that miR-133b expression in medial longitudinal fascicle, superior reticular formation, and intermediate reticular formation neurons is essential for full locomotor recovery after a spinal cord injury. During retinal regeneration, dedifferentiation of Müller glia into a cycling population of progenitor cells enables the injured retina to restore its function (Ramachandran et al. 2010). This process is partially stimulated by *ascl1a* regulation of Lin-28 protein, which decreases let-7 level. Lin-28 and let-7 have inverse regulatory link (Rybak et al. 2008). Thus, inhibition of let-7 promotes the expression of genes that are necessary for retina regeneration in zebrafish (Ramachandran et al. 2010).

The mechanisms of regeneration are complex and involve multiple signaling molecules, transcriptional factors, and genes. So far, few miRNA targets have been validated. Although many pathways have shown overarching conservation among the regenerative tissues, further exploration of tissue specificity and conservation of miRNAs among the regenerative organs may provide better knowledge of regenerative mechanisms.

## miRNA in Teleost Reproduction

### Oogenesis and Spermatogenesis

Studies in mammals indicate that miRNAs are crucial in oogenesis and spermatogenesis (Takada et al. 2009; Yadav and Kotaja 2014). The balance between self-renewal and differentiation of spermatogonial cells is critical in seasonally reproducing fishes to define the start and the end of reproductive activity; thus, the maintenance of this balance requires posttranscriptional regulation of a number of genes. However, no information is available on miRNA functions in the spermatogonial phase of teleost spermatogenesis. In miRNA microarray experiment, differential expression of 13 miRNAs was found at previtellogenesis, vitellogenesis, late vitellogenesis, and maturation stages during oogenesis in rainbow trout. miRNA targets, important in oocyte maturation, growth, development, and maturational competence, that is the ability of oocyte to resume meiosis, were also predicted (Juanchich et al. 2013). However, this and other few studies on miRNA in teleost gonadal development (Bizuayehu et al. 2012b; Abramov et al. 2013) give an insight into miRNA developmental profile rather than decipher cell type-specific functions.

Many circulating endocrine and locally acting paracrine and autocrine factors regulate oogenesis and spermatogenesis (Lubzens et al. 2010; Schulz et al. 2010). Estrogens are important in teleost spermatogonial self-renewal and in the transition of type A to type B spermatogonia (Schulz et al. 2010). There is a complex interplay between estrogens and miRNAs. Estrogens regulate transcription of some miRNAs, such as miR-21 and miR-221; in contrast, other miRNAs such as let-7, miR-22, miR-196b, or miR-206 target estrogen receptor alpha transcript (Cochrane et al. 2011). In mammals, miR-383 regulates 17β-estradiol release from granulosa cells (Yin et al. 2012). Also androgens regulate the expression of some miRNAs, such as miR-22, miR-122, and mir-125b (Cochrane et al. 2011); however, direct posttranscriptional regulation of androgens by miRNAs is not elucidated yet. The effect of masculinization treatment with either a synthetic androgen (17-α-methyl testosterone) or an inhibitor of cytochrome P450 aromatase (Fadrozole) on miRNA expression has been studied in Atlantic halibut; masculinization treatment resulted in differential expression of let-7a, miR-19b, miR-24, and miR-202-3p in gonads (Bizuayehu et al. 2012b). Future work is needed to investigate the role of miRNAs in hormone-secreting gonadal cells.

### Sexual Maturation

Control of puberty is an important issue in fish farming. Precocious sexual maturation has economic implications, and various methods have been devised to attain the efficient production and public acceptance in terms of welfare and sustainability (Taranger et al. 2010). Selective breeding, environmental manipulation, induced triploidy, and monosex production are among the methods used to control the sexual maturation. The brain–pituitary–gonad axis regulates sexual maturation in teleosts. Precise mechanisms are not fully understood. Several factors influence sexual maturation, including habitat, temperature, photoperiod, nutritional status, social interaction, pheromones, and hormones, all having role in the extensive regulatory feedback system (Taranger et al. 2010).

Little is known about the role of miRNAs during sexual maturation in teleosts. miRNA profiling studies in Atlantic halibut showed significant differences in the expression of many miRNAs. The expression of let-7a, miR-143, miR-145, and miR-202-3p was significantly higher in adult testis compared with adult ovary, and miR-451 was significantly downregulated in brain of juveniles compared with adult females (Bizuayehu et al. 2012b). In Nile tilapia, the expression of miR-129-3p and miR-727-3p was significantly higher in mature females than males, whereas the expression of miR-132a and miR-212 was significantly higher in mature males than females (Xiao et al. 2014). Differential expression of miRNAs between mature and immature gonads has been reported in rat (Gaytan et al. 2013), pig (Luo et al. 2010), and chicken (Kang et al. 2013). Change in expression of miR-145/c-Myc/Lin-28/let-7 axis in hypothalamus occurs during rat puberty (Gaytan et al. 2013). In rainbow trout, 13 miRNAs showed differential expression patterns during the ovarian development (Juanchich et al. 2013). Further research on mechanisms involved in the regulation of sexual maturation through epigenetics, and miRNA-related pathways would advance our understanding of fish sexual development.

## miRNA in Endocrine Organs of Teleosts

Hormone-secreting organs in teleosts include the brain (hypothalamus, pituitary, and pineal gland), thyroid, kidney (chromaffin tissue and corpuscles of stannous), gonad (theca and Leydig cells), intestinal mucosa, pancreatic islets, ultimobranchial body, and urophysis. The development and tissue specification of these organs involve miRNAs. Expression profiling studies in fish indicate tissue specificity of miRNAs in some of these organs (Wienholds et al. 2005; Kapsimali et al. 2007; Kloosterman et al. 2007; Tessmar-Raible et al. 2007; Bizuayehu et al. 2012b).

### Brain

Endocrine compartments of the brain: Hypothalamus, pituitary, and pineal gland are involved in the regulation of homeostasis.

#### The Hypothalamus

The hypothalamus comprised preopticus, lateralis tuberis, recessus lateralis, and recessus posterioris nuclei, which penetrate the pituitary to promote hormonal releases (Goos 1978). Few studies show expression of miRNAs in hypothalamus of teleosts (Wienholds et al. 2005; Tessmar-Raible et al. 2007). Some of these miRNAs, such as miR-7 and miR-7b, are found both in mice (Bak et al. 2008) and zebrafish (Tessmar-Raible et al. 2007), indicating probable functional conservation. Although there is no report available on miRNA expression in different nuclei in teleost hypothalamus, miRNA profiling of mammalian homologous regions indicates differential and specific expression patterns (Herzer et al. 2012). Corticotrophin-releasing factor from hypothalamus enhances proopiomelanocortin transcription and promotes adrenocorticotropic hormone production. This process is negatively regulated by miR-375 in mice (Zhang et al. 2013a). Another corticotrophin-releasing factor, urocortin 2, promotes expression of miR-325-3p in pituitary and suppresses biosynthesis and secretion of luteinizing hormone in rat (Nemoto et al. 2012). Gonadotropin-releasing hormone regulates multiple miRNA expression in gonadotrope cell lines, producing downregulation of miR-99b and miR-125b, and upregulation of miR-132, miR-151, miR-212, miR-222, miR-350, and miR-424 (Godoy et al. 2011). Intronic miR-132 and miR-212 target p250RhoGAP, thereby guide morphological change and increase the motility of gonadotropes (Godoy et al. 2011). In chicken, hypothalamic neuronal cell migration is mediated through miR-138 repression of *RELN* (Kisliouk and Meiri 2013).

#### The Pituitary Gland

One of the functions of the pituitary gland is signal transmission between hypothalamus and peripheral tissues. In teleosts, pituitary is composed of neurohypophysis, a neural component originated from the diencephalon, and adenohypophysis derived from the buccal epithelium (Schreibman et al. 1973). Adenohypophysis is divided into rostral pars distalis or proadenohypophysis, proximal pars distalis or meso-adenohypophysis, and pars intermedia or meta-adenohypophysis (Schreibman et al. 1973). There are many signaling molecules and transcriptional factors that control teleost pituitary development and patterning (Sbrogna et al. 2003; Herzog et al. 2004; Nica et al. 2006). Some of these transcriptional factors are known to be targeted by miRNAs in mammals; for example, miR-26b represses lymphoid enhancer factor 1 to promote the generation of somatotrope, lactotrope, and thyrotrope cell lineages in mice (Zhang et al. 2010). In contrast to mammals, there are interdigitations between neurohypophysis and adenohypophysis in teleosts. Considering the role of miRNAs in defining boundaries, such as midhind brain boundary (Leucht et al. 2008) and hindbrain and spinal cord boundary (Woltering and Durston 2008), the presence of functional distinction in amalgamated tissues raises the question whether miRNAs have a role in demarcation of the pituitary morphological boundaries. Teleosts rostral pars distalis is composed of various cell types, such as η, ϵ, neck, and channel cells (Schreibman et al. 1973). η and ϵ cells produce prolactin and adrenocorticotrophic hormone, respectively, whereas other cells are involved in the movement of material into or from the follicular lumina, as well as in phagocytosis and dispersal of a hormone or a carrier substance into peripheral circulation (Schreibman et al. 1973; Leatherland 1976). Teleost proximal pars distalis is composed of cells secreting thyrotropins, somatotropins and gonadotropins, whereas pars intermedia contains two cell types, which secrete melanocyte stimulating hormone and somatolactin (Rand-Weaver et al. 1991; Schreibman et al. 1973). Differentiation and functional specification of these cells require specific regulatory elements promoting (Liu et al. 2003) or inhibiting (Liu et al. 2006) lineage-specific gene expression. The role of miRNAs in such the specification and in release of neurosecretory material remains unknown. In mammals, miR-7, miR-7b, miR-141, miR-200a, and miR-375 are enriched in the pituitary (Landgraf et al. 2007; Bak et al. 2008); similarly, in situ detection during zebrafish development shows the expression of miR-375 in pituitary (Wienholds et al. 2005; Kapsimali et al. 2007), indicating evolutionary conservation of miRNA in pituitary function.

#### The Pineal Gland

The pineal gland modulates physiological activities related to daily and seasonal rhythmicity in fish. Production of the main compound, melatonin, is stimulated by darkness and inhibited by light. Involvement of miRNAs in teleost pineal gland development and secretion is unknown. In rat pineal gland, expression of several dominant miRNAs such as miR-96, miR-124, miR-125b, miR-127, miR-182, and miR-183 has been found (Clokie et al. 2012). Although majority of miRNAs identified by the authors had similar expression during the day and night, the 2-fold increase in miR-96, miR-182, and miR-183 during the day when compared with the night was found. Moreover, a significant reduction of melatonin in miR-483-transfected pinealocytes and repression of arylalkylamine *N*-acetyltransferase expression, an enzyme converting serotonin to N-acetylserotonin, was observed. It is essential to consider the differences in photoperiodic and circadian control of neuroendocrine functions between teleosts and mammals. The mammalian system has a linear flow (eye–hypothalamus–pineal gland) to the rhythmic production of melatonin, whereas in fish, melatonin biosynthesis and degradation require synchronized complex interactions among eye, pineal gland, brain (particularly hypothalamus and pituitary), and peripheral tissues upon the stimulation with light (Falcón et al. 2010). Therefore, functional characterization of miRNAs in teleost pineal gland will be important to understand neuroendocrine regulation of rhythmicity by the pineal gland.

### The Thyroid Gland

The thyroid gland secretions, thyroid hormones (THs), are conserved among vertebrates. They mediate gene expression by binding to thyroid-hormone-binding protein, which binds to TH responsive element and regulates the transcription of different genes (Wu and Koenig 2000). In teleosts, THs are maternally deposited in eggs (Brooks et al. 1997). They are essential for growth and metabolism throughout the life (Power et al. 2001; Liu and Chan 2002) and regulate different aspects of development including metamorphosis, which requires substantial changes in morphology, physiology, and behavior (Yamano et al. 1991; Liu and Chan 2002). Metamorphosis is accompanied by alteration in miRNA profile (Fu et al. 2011, 2013; Bizuayehu et al. 2012a). In rat thyroid cells, many miRNAs such as miR-1, miR-28a, and miR-296-3p are differentially expressed and possibly they target transcripts that are important in thyroid cell proliferation (Leone et al. 2011). Akama et al. (2012) demonstrated that the expression of 47 miRNAs in rat thyroid cells was reduced after the addition of thyroid stimulating hormone. Their result suggests that thyroid stimulating hormone regulates thyroid cell proliferation partly by reducing the expression of miR-16 and miR-195, which target important cell proliferation genes, including *Mapk8, Ccne1,* and *Cdc6*. Similarly, using Dicer conditional knock-out model, Frezzetti et al. (2011) showed the importance of miRNAs in the morphology and function of thyroid gland in mice.

### The Endocrine Pancreas

The endocrine pancreas physiology has been studied in fishes for more than a century. Pancreatic hormones: Insulin, glucagon, glucagon-like peptide, somatostatin, pancreastatin, and pancreatic peptide have been isolated and localized in different cells of the pancreas (Plisetskaya 1989; Jonsson 1991). The secretion of pancreatic hormones is a tightly controlled process with feedback mechanisms. Little is known about the physiological function of miRNAs in teleost pancreas (Wienholds et al. 2005; Kloosterman et al. 2007). Some miRNAs, such as miR-375, are uniquely expressed in pancreatic cells; Kloosterman et al. (2007) have shown the importance of miR-375 in the insulin-secreting pancreatic islets, as miR-375-knockdown zebrafish embryos had dispersed islet cells. In mammals, miR-7, miR-9, miR-29b, miR-30d, miR-124a, and miR-375 regulate the secretion and islet development (Poy et al. 2004; Baroukh and Van Obberghen 2009; Tang et al. 2009; Pullen et al. 2011). Poy et al. (2004) have demonstrated that miR-375 targets myotrophin and is involved in insulin exocytosis. Similarly, Plaisance et al. (2006) have reported the control of the secretory function of insulin-producing cells by miR-9. In their electrophoretic mobility shift assay, chromatine immunoprecipitation, and gene reporter experiments, the transcriptional factor onecut-2, which targets granuphilin, is implicated in insulin secretion, and its level is kept at an appropriate level by miR-9. Given a persistent hyperglycemia in some aquaculture species fed carbohydrate-rich diets (Moon 2001), studies on carbohydrate metabolism in the context of gene regulation may help in understanding the consequences of fish meal replacement with plant products in aquaculture feeds. Therefore, a catalog of abundant and specific miRNAs in pancreatic cells of teleosts and comparison of expression patterns and types of miRNAs among different phylogenetic groups will advance our understanding of endocrine pancreas in teleosts.

### Other Endocrine Cells/Tissues

There are no reports yet on miRNA characterization in other endocrine tissues of teleosts, including chromaffin tissue, cortical tissue, hormone secretory cells in gonad, intestinal mucosa, or urophysis.

## Response to Environment

### Osmoregulation

Osmoregulation in teleosts is a process whereby an organism adapts to different ionic environments. In zebrafish embryos, miR-8 family (miR-8, miR-141, miR-200a, miR-200b, miR-200c, and miR-429) has been expressed in ionocytes (Flynt et al. 2009), which are specialized branchial epithelial cells that are involved in the maintenance of osmotic homeostasis. An increase in osmotic stress sensitivity of miR-200a and miR-200b morphants was observed along with Na^+^ accumulation in ionocytes, indicating the function of miR-8 family in Na^+^/H^+^ exchanger (2009). In adult Nile tilapia, apart from high expression of miR-30 family and miR-429 in kidney and gills, miR-30c and miR-429 are implicated in osmotic stress regulation (Yan, Zhao, et al. 2012; Yan et al. 2012b). It is not known whether the different cells that are involved in osmoregulation respond in similar fashion, what is cell:miRNAs specificity, and whether similar miRNAs are involved in osmoregulation in marine and freshwater species. Participation of miRNAs in osmotic stress regulation is disclosed through the modulation of genes that are involved in regulation of membrane dynamics, trafficking of transmembrane proteins, and osmoregulatory signaling, such as Na^+^/H^+^ exchanger regulatory factor 1 in zebrafish (Flynt et al. 2009) and osmotic stress transcriptional factor 1 in Nile tilapia (Yan, Zhao, et al. 2012). Considering functions of different hormones, such as growth hormone, somatolactin and prolactin in ion control in teleost fish (Rand-Weaver et al. 1991), a large regulatory pathway model can be anticipated. Elucidating miRNA functions in passive and active transporters through the regulation of membrane-bound proteins, peripheral proteins, and/or porin structures would help to understand osmoregulatory mechanisms.

### Temperature

Temperature is a major external determinant of teleost physiology. All teleosts have a preferred water temperature, and a deviation from the optimum range can affect survival, growth, behavior, and reproduction. miRNAs have important role in regulation of environmental effects (Li et al. 2009); however, little is known about their role in teleosts thermal regulation. Although 11 upregulated and 15 downregulated miRNAs were found in brain of cold-acclimated zebrafish, their overall contribution in the regulation of protein-coding genes was found to be minimal (Yang et al. 2011). The authors suggested that brain miRNAs had developmental rather than thermal adaptation functions. More work is needed to test this hypothesis using different species, tissues, and experimental set-ups. Significant differences in miRNA expression between hyperplasic myotube and hypertrophic phenotypes have been found in zebrafish as the effect of temperature in early development (Johnston et al. 2009). This report also showed significant differences in myotomal fast muscle fiber recruitment in embryos held at different incubation temperatures (22 °C, 26 °C, and 31 °C). Thus, it is interesting to know the role of miRNA in this process. Further work on both eurythermal and stenothermal teleosts is needed to understand the functions of miRNAs in thermal regulation. It is important in the context of global warming in general and rise in oceans temperature in particular. Knowledge on the miRNA regulatory modulation as a function of temperature can be useful to model ecological and biological consequences of the global warming.

### Stress

Transcriptional and posttranscriptional adjustments of gene expression during the stress are among the long-term responses, and they are primarily regulated by hormones (Fiol and Kültz 2007). The accumulation of Argonaute proteins and miRNAs in separate cytoplasmic foci in stress-dependent manner (Leung et al. 2006) indicates possible modulation of stress response by miRNAs. Different stressors, such as oxidative, osmotic, or heat shock can alter miRNA expression.

#### Oxidative Stress

Oxidative stress is caused by reactive oxygen species and has a potential to inhibit cellular functions, but natural antioxidants such as tocopherols (vitamin E) can limit the damage. Feeding Nile tilapia a diet containing various levels of vitamin E (0, 50, and 2,500 mg/kg) resulted in reduction in the expression of miR-16, miR-122, miR-146a and miR-223 in E-deficient group and increased the expression of miR-16, miR-21, miR-122, miR-125b, miR-146a, miR-155, miR-181a, and miR-223 in E-enriched group, when compared with E-normal group (Tang et al. 2013). Yu et al. (2010) have shown antioxidant activity of miR-451 in zebrafish embryos. Further investigations on the role of miRNAs in oxidative stress and their potential as biomarkers are important for aquaculture to improve welfare and growth performance of fish.

#### Osmotic Stress

Uptake of hyperosmotic 2% saline water resulted in upregulation of expression of miR-7b, miR-9, miR-29b, miR-137, and miR-451 and downregulation of miR-409, miR-107, miR-103, miR-185, and miR-320 in hypothalamus in mice (Lee et al. 2006). This study showed also a reduction of Fos protein production (component of a transcriptional factor activator protein 1) in paraventricular nucleus as the result of miR-7b activity. Similarly, salt tolerance of Nile tilapia is partly attributed to regulatory function of some miRNAs, such as miR-30c and miR-429 (Yan, Zhao, et al. 2012; Yan et al. 2012b). Nevertheless, the mechanism of miRNA action in osmotic stress response is not known yet.

#### Irradiation, Chemical, and Physical Stressors

In zebrafish embryos, miR-125b is downregulated by gamma-irradiation and upon exposure to a cytotoxic chemical, which corresponds to an increase in p53 protein level; it indicates the possible function of miR-125b in stress response regulation (Le et al. 2009). Physical stress can act as a “morphogen”; for example, shear stress and stretch of cardiomyocytes activate the differential expression of miR-21 during heart valve formation in zebrafish (Banjo et al. 2013). Exposure of lake whitefish (*Coregonus lavaretus*) to microcystin significantly changed the expression of 6 liver miRNAs in a time-dependent manner (Brzuzan et al. 2012), indicating the involvement of miRNAs at different levels of physiological acclimation responses. Likewise, 24 h exposure of adult zebrafish to different concentrations of microcystins altered the expression of 4 miRNAs (Li et al. 2013). Although the extract, delivery method, and concentration differed in those two reports, miR-122 expression pattern was opposite in whitefish and zebrafish liver at the similar exposure time. Exposure to other chemicals, such as T2,3,7,8-tetrachlorodibenzo-p-dioxin and perfluorooctane sulfonate also altered the expression of group of miRNAs during the zebrafish development (Zhang et al. 2011; Jenny et al. 2012). All these studies indicate that miRNA expression is affected by a variety of environmental stressors; however, the mechanisms of this modulation and their consequences need to be further elucidated.

## In Vivo Models for miRNA Studies in Teleosts

In vivo models are useful tools for expanding knowledge about the molecular mechanisms. They are not widely used yet in miRNA research in teleosts.

The first teleosts cell line, RTG2, has been derived from a gonad of rainbow trout (Wolf and Quimby 1962). Since then a number of fish cell lines have been established and utilized in many fields of biological research. A rainbow trout spleen cell line, RTS34, was used in target validation of a miRNA in Atlantic halibut, which confirmed the binding of miR-24 to the 3′-UTR of kiss peptin 1 receptor-2 (Bizuayehu et al. 2013).

Apart from in vitro studies, miRNA mutant and transgenic lines should be explored as these lines in zebrafish have widened our understanding of the molecular processes that govern the phenotypic outputs of several transcriptional factors, signaling molecules, and genes. However, functional versatility of miRNAs during the transcription and posttranscription processes suggest careful reexamination of the existing knock-out/mutants models in terms of miRNA function.

Recent genome editing technologies, such as zinc finger nucleases, transcription activator-like effector nucleases (TALENs), CRISP-Cas, or RNA-guided Cas9 nuclease (Urnov et al. 2010; Bedell et al. 2012; Chang et al. 2013; Cong et al. 2013; Hwang et al. 2013) provide an opportunity to edit a specific miRNA or a cluster in the genome. For example, two miRNA clusters, miR-17-92 and miR-430, were successfully interrupted in zebrafish using TALEN technology (Liu et al. 2013). The functional characterization of miRNAs will be accelerated by integrating these methods with spatiotemporal miRNA transcript regulation (knock-down and overexpression) using various methods such as caged antagomirs (Connelly et al. 2012; Griepenburg et al. 2013). The prospect in this regard is immense.

## Conclusions

Functions of miRNAs in teleost development, growth, and physiology are not satisfactory understood yet. Evolutionary diversity of teleosts and the resulting plasticity in their environmental adaptation suggest that there is much more to discover beyond the very few species and processes investigated so far. This review indicates the importance of functional studies. Although a given miRNA may be either predicted (in silico), or demonstrated (in situ), to have a regulatory capacity, its physiological relevance has to be established in a specific context of a live system. Characterization and profiling of miRNAs in different teleosts is the beginning of a long road ahead to disclose the functional conservation and variations of miRNA regulatory pathways across Teleostei.

## References

[evu151-B1] Abramov R, Fu G, Zhang Y, Peng C (2013). Expression and regulation of miR-17a and miR-430b in zebrafish ovarian follicles. Gen Comp Endocrinol..

[evu151-B2] Akama T (2012). Identification of microRNAs that mediate thyroid cell growth induced by TSH. Mol Endocrinol..

[evu151-B3] Almeida FFL, Kristoffersen C, Taranger GL, Schulz RW (2008). Spermatogenesis in Atlantic cod (*Gadus morhua*): a novel model of cystic germ cell development. Biol Reprod..

[evu151-B4] Andreassen R, Worren M, Hoyheim B (2013). Discovery and characterization of miRNA genes in Atlantic salmon (*Salmo salar*) by use of a deep sequencing approach. BMC Genomics.

[evu151-B5] Ason B (2006). Differences in vertebrate microRNA expression. Proc Natl Acad Sci U S A..

[evu151-B6] Babiarz JE, Ruby JG, Wang Y, Bartel DP, Blelloch R (2008). Mouse ES cells express endogenous shRNAs, siRNAs, and other microprocessor-independent, Dicer-dependent small RNAs. Genes Dev..

[evu151-B7] Bak M (2008). MicroRNA expression in the adult mouse central nervous system. RNA.

[evu151-B8] Banjo T (2013). Haemodynamically dependent valvulogenesis of zebrafish heart is mediated by flow-dependent expression of miR-21. Nat Commun..

[evu151-B9] Barckmann B, Simonelig M (2013). Control of maternal mRNA stability in germ cells and early embryos. Biochim Biophys Acta..

[evu151-B10] Bardon A (2009). What is the heritable component of spinal deformities in the European sea bass (*Dicentrarchus labrax*)?. Aquaculture.

[evu151-B11] Baroukh NN, Van Obberghen E (2009). Function of microRNA-375 and microRNA-124a in pancreas and brain. FEBS J..

[evu151-B12] Barozai MYK (2012). Identification and characterization of the microRNAs and their targets in *Salmo salar*. Gene.

[evu151-B13] Bartel D (2009). MicroRNAs: target recognition and regulatory functions. Cell.

[evu151-B14] Bartel DP (2004). MicroRNAs: genomics, biogenesis, mechanism, and function. Cell.

[evu151-B15] Bedell VM (2012). In vivo genome editing using a high-efficiency TALEN system. Nature.

[evu151-B16] Bekaert M (2013). Sequencing and characterisation of an extensive Atlantic salmon (*Salmo salar* L.) microRNA repertoire. PLoS One.

[evu151-B17] Bennasser Y (2011). Competition for XPO5 binding between Dicer mRNA, pre-miRNA and viral RNA regulates human Dicer levels. Nat Struct Mol Biol..

[evu151-B18] Biyashev D (2012). miR-27b controls venous specification and tip cell fate. Blood.

[evu151-B19] Bizuayehu TT, Fernandes JMO, Johansen SD, Babiak I (2013). Characterization of novel precursor mirnas using next generation sequencing and prediction of miRNA targets in Atlantic halibut. PLoS One.

[evu151-B20] Bizuayehu TT (2012a). Differential expression patterns of conserved miRNAs and isomiRs during Atlantic halibut development. BMC Genomics.

[evu151-B21] Bizuayehu TT (2012b). Sex-biased miRNA expression in Atlantic halibut (*Hippoglossus hippoglossus*) brain and gonads. Sex Dev..

[evu151-B22] Blum N, Begemann G (2012). Retinoic acid signaling controls the formation, proliferation and survival of the blastema during adult zebrafish fin regeneration. Development.

[evu151-B23] Borchert GM, Lanier W, Davidson BL (2006). RNA polymerase III transcribes human microRNAs. Nat Struct Mol Biol..

[evu151-B24] Bortolin-Cavaille M-L, Dance M, Weber M, Cavaille J (2009). C19MC microRNAs are processed from introns of large Pol-II, non-protein-coding transcripts. Nucleic Acids Res..

[evu151-B25] Brainerd EL, Slutz SS, Hall EK, Phillis RW (2001). Patterns of genome size evolution in tetraodontiform fishes. Evolution.

[evu151-B26] Brodersen P, Voinnet O (2009). Revisiting the principles of microRNA target recognition and mode of action. Nat Rev Mol Cell Biol..

[evu151-B27] Brooks S, Tyler C, Sumpter J (1997). Egg quality in fish: what makes a good egg?. Rev Fish Biol Fisheries..

[evu151-B28] Brunet FG (2006). Gene loss and evolutionary rates following whole-genome duplication in teleost fishes. Mol Biol Evol..

[evu151-B29] Brzuzan P, Woźny M, Wolińska L, Piasecka A (2012). Expression profiling in vivo demonstrates rapid changes in liver microRNA levels of whitefish (*Coregonus lavaretus*) following microcystin-LR exposure. Aquatic Toxicol..

[evu151-B30] Burroughs AM, Kawano M, Ando Y, Daub CO, Hayashizaki Y (2011). pre-miRNA profiles obtained through application of locked nucleic acids and deep sequencing reveals complex 5′/3′ arm variation including concomitant cleavage and polyuridylation patterns. Nucleic Acids Res..

[evu151-B31] Burroughs AM (2010). A comprehensive survey of 3' animal miRNA modification events and a possible role for 3' adenylation in modulating miRNA targeting effectiveness. Genome Res..

[evu151-B32] Cai XZ, Hagedorn CH, Cullen BR (2004). Human microRNAs are processed from capped, polyadenylated transcripts that can also function as mRNAs. RNA.

[evu151-B33] Camarata T (2010). Pdlim7 (LMP4) regulation of Tbx5 specifies zebrafish heart atrio-ventricular boundary and valve formation. Dev Biol..

[evu151-B34] Cavodeassi F (2005). Early stages of zebrafish eye formation require the coordinated activity of Wnt11, Fz5, and the Wnt/β-catenin pathway. Neuron.

[evu151-B35] Chang N (2013). Genome editing with RNA-guided Cas9 nuclease in zebrafish embryos. Cell Res..

[evu151-B36] Chaturvedi A, Raeymaekers JAM, Volckaert FAM (2014). Computational identification of miRNAs, their targets and functions in three-spined stickleback (*Gasterosteus aculeatus*). Mol Ecol Resour..

[evu151-B37] Chauvigne F, Cauty C, Ralliere C, Rescan PY (2005). Muscle fiber differentiation in fish embryos as shown by *in situ* hybridization of a large repertoire of muscle-specific transcripts. Dev Dyn..

[evu151-B38] Chaves-Pozo E, Mulero V, Meseguer J, García Ayala A (2005). An overview of cell renewal in the testis throughout the reproductive cycle of a seasonal breeding teleost, the gilthead seabream (*Sparus aurata* L). Biol Reprod..

[evu151-B39] Cheloufi S, Dos Santos CO, Chong MMW, Hannon GJ (2010). A Dicer-independent miRNA biogenesis pathway that requires Ago catalysis. Nature.

[evu151-B40] Chen J-F (2006). The role of microRNA-1 and microRNA-133 in skeletal muscle proliferation and differentiation. Nat Genet..

[evu151-B41] Chen PY (2005). The developmental miRNA profiles of zebrafish as determined by small RNA cloning. Genes Dev..

[evu151-B42] Chi SW, Hannon GJ, Darnell RB (2012). An alternative mode of microRNA target recognition. Nat Struct Mol Biol..

[evu151-B43] Chiang HR (2010). Mammalian microRNAs: experimental evaluation of novel and previously annotated genes. Genes Dev..

[evu151-B44] Chiavacci E (2012). *MicroRNA-218* mediates the effects of *Tbx5a* over-expression on zebrafish heart development. PLoS One.

[evu151-B45] Choudhury NR (2013). Tissue-specific control of brain-enriched miR-7 biogenesis. Genes Dev..

[evu151-B46] Chow RL, Lang RA (2001). Early eye development in vertebrates. Annu Rev Cell Dev Biol..

[evu151-B47] Clokie SJH, Lau P, Kim HH, Coon SL, Klein DC (2012). MicroRNAs in the Pineal Gland miR-483 regulates melatonin synthesis by targeting arylalkylamine N-acetyltransferase. J Biol Chem..

[evu151-B48] Cloonan N (2011). MicroRNAs and their isomiRs function cooperatively to target common biological pathways. Genome Biol..

[evu151-B49] Clop A (2006). A mutation creating a potential illegitimate microRNA target site in the myostatin gene affects muscularity in sheep. Nat Genet..

[evu151-B50] Cochrane DR, Cittelly DM, Richer JK (2011). Steroid receptors and microRNAs: relationships revealed. Steroids.

[evu151-B51] Cong L (2013). Multiplex genome engineering using CRISPR/Cas systems. Science.

[evu151-B52] Connelly CM, Uprety R, Hemphill J, Deiters A (2012). Spatiotemporal control of microRNA function using light-activated antagomirs. Mol Biosyst..

[evu151-B53] Conte I (2010). miR-204 is required for lens and retinal development via Meis2 targeting. Proc Natl Acad Sci U S A..

[evu151-B54] Crow KD, Stadler PF, Lynch VJ, Amemiya C, Wagner GP (2006). The “fish-specific” Hox cluster duplication is coincident with the origin of teleosts. Mol Biol Evol..

[evu151-B55] De Pater E (2009). Distinct phases of cardiomyocyte differentiation regulate growth of the zebrafish heart. Development.

[evu151-B56] Doitsidou M (2002). Guidance of primordial germ cell migration by the chemokine SDF-1. Cell.

[evu151-B57] Donadeu FX, Schauer SN, Sontakke SD (2012). Involvement of miRNAs in ovarian follicular and luteal development. J Endocrinol..

[evu151-B58] Dueck A, Meister G (2010). MicroRNA processing without Dicer. Genome Biol..

[evu151-B59] Dunworth WP (2013). Bone morphogenetic protein 2 signaling negatively modulates lymphatic development in vertebrate embryos. Circ Res..

[evu151-B60] Duszynski RJ, Topczewski J, Leclair EE (2013). Divergent requirements for fibroblast growth factor signaling in zebrafish maxillary barbel and caudal fin regeneration. Dev Growth Differ..

[evu151-B61] Eberhart JK (2008). MicroRNA Mirn140 modulates Pdgf signaling during palatogenesis. Nat Genet..

[evu151-B62] Elworthy S, Hargrave M, Knight R, Mebus K, Ingham PW (2008). Expression of multiple slow myosin heavy chain genes reveals a diversity of zebrafish slow twitch muscle fibres with differing requirements for Hedgehog and Prdm1 activity. Development.

[evu151-B63] Eulalio A, Huntzinger E, Izaurralde E (2008). Getting to the root of miRNA-mediated gene silencing. Cell.

[evu151-B64] Fabian MR, Sonenberg N (2012). The mechanics of miRNA-mediated gene silencing: a look under the hood of miRISC. Nat Struct Mol Biol..

[evu151-B65] Fabian MR (2009). Mammalian miRNA RISC recruits CAF1 and PABP to affect PABP-dependent deadenylation. Mol Cell..

[evu151-B66] Fadool JM, Dowling JE (2008). Zebrafish: a model system for the study of eye genetics. Prog Ret Eye Res..

[evu151-B67] Falcón J, Migaud H, Muñoz-Cueto JA, Carrillo M (2010). Current knowledge on the melatonin system in teleost fish. Gen Comp Endocrinol..

[evu151-B68] Fang Z, Rajewsky N (2011). The impact of miRNA target sites in coding sequences and in 3′UTRs. PLoS One.

[evu151-B69] Fernandez-Valverde SL, Taft RJ, Mattick JS (2010). Dynamic isomiR regulation in *Drosophila* development. RNA.

[evu151-B70] Finnegan EF, Pasquinelli AE (2013). MicroRNA biogenesis: regulating the regulators. Crit Rev Biochem Mol Biol..

[evu151-B71] Fiol DF, Kültz D (2007). Osmotic stress sensing and signaling in fishes. FEBS J..

[evu151-B72] Fish JE (2008). miR-126 regulates angiogenic signaling and vascular integrity. Dev Cell..

[evu151-B73] Fish JE (2011). A Slit/miR-218/Robo regulatory loop is required during heart tube formation in zebrafish. Development.

[evu151-B74] Flores MV, Lam EYN, Crosier P, Crosier K (2006). A hierarchy of Runx transcription factors modulate the onset of chondrogenesis in craniofacial endochondral bones in zebrafish. Dev Dyn..

[evu151-B75] Flynt AS, Li N, Thatcher EJ, Solnica-Krezel L, Patton JG (2007). Zebrafish miR-214 modulates Hedgehog signaling to specify muscle cell fate. Nat Genet..

[evu151-B76] Flynt AS (2009). miR-8 microRNAs regulate the response to osmotic stress in zebrafish embryos. J Cell Biol..

[evu151-B77] Frezzetti D (2011). The microRNA-processing enzyme Dicer is essential for thyroid function. PLoS One.

[evu151-B78] Friedman RC, Farh KK-H, Burge CB, Bartel DP (2009). Most mammalian mRNAs are conserved targets of microRNAs. Genome Res..

[evu151-B79] Fu Y (2013). Expression of let-7 microRNAs that are involved in Japanese flounder (*Paralichthys olivaceus*) metamorphosis. Comp Biochem Physiol B Biochem Mol Biol..

[evu151-B80] Fu YS (2011). Identification and differential expression of microRNAs during metamorphosis of the Japanese flounder (*Paralichthys olivaceus*). PLoS One.

[evu151-B81] Fujimoto T (2010). Sexual dimorphism of gonadal structure and gene expression in germ cell-deficient loach, a teleost fish. Proc Natl Acad Sci U S A..

[evu151-B82] Fukuda T (2007). DEAD-box RNA helicase subunits of the Drosha complex are required for processing of rRNA and a subset of microRNAs. Nat Cell Biol..

[evu151-B83] Gantier MP (2011). Analysis of microRNA turnover in mammalian cells following Dicer1 ablation. Nucleic Acids Res..

[evu151-B84] García-López J, Hourcade JDD, Del Mazo J (2013). Reprogramming of microRNAs by adenosine-to-inosine editing and the selective elimination of edited microRNA precursors in mouse oocytes and preimplantation embryos. Nucleic Acids Res..

[evu151-B85] Gaytan F (2013). Distinct expression patterns predict differential roles of the mirna-binding proteins, lin28 and lin28b, in the mouse testis: studies during postnatal development and in a model of hypogonadotropic hypogonadism. Endocrinology.

[evu151-B86] Georges M, Coppieters W, Charlier C (2007). Polymorphic miRNA-mediated gene regulation: contribution to phenotypic variation and disease. Curr Opin Genet Dev..

[evu151-B87] Gestri G, Link BA, Neuhauss SCF (2012). The visual system of zebrafish and its use to model human ocular diseases. Dev Neurobiol..

[evu151-B88] Ghildiyal M, Zamore PD (2009). Small silencing RNAs: an expanding universe. Nat Rev Genet..

[evu151-B89] Giraldez AJ (2005). MicroRNAs regulate brain morphogenesis in zebrafish. Science.

[evu151-B90] Giraldez AJ (2006). Zebrafish MiR-430 promotes deadenylation and clearance of maternal mRNAs. Science.

[evu151-B91] Godoy J, Nishimura M, Webster NJG (2011). Gonadotropin-releasing hormone induces miR-132 and miR-212 to regulate cellular morphology and migration in immortalized LβT2 pituitary gonadotrope cells. Mol Endocrinol..

[evu151-B92] Goljanek-Whysall K (2011). MicroRNA regulation of the paired-box transcription factor Pax3 confers robustness to developmental timing of myogenesis. Proc Natl Acad Sci U S A..

[evu151-B93] Goos HJT (1978). Hypophysiotropic centers in the brain of amphibians and fish. Am Zool..

[evu151-B94] Griepenburg JC, Ruble BK, Dmochowski IJ (2013). Caged oligonucleotides for bidirectional photomodulation of let-7 miRNA in zebrafish embryos. Bioorg Med Chem..

[evu151-B95] Griffiths-Jones S, Saini HK, Van Dongen S, Enright AJ (2008). miRBase: tools for microRNA genomics. Nucleic Acids Res..

[evu151-B96] Grimes AC (2010). Phylogeny informs ontogeny: a proposed common theme in the arterial pole of the vertebrate heart. Evol Dev..

[evu151-B97] Gu T (2012). Canonical A-to-I and C-to-U RNA editing is enriched at 3′UTRs and microRNA target sites in multiple mouse tissues. PLoS One.

[evu151-B98] Guo H, Ingolia NT, Weissman JS, Bartel DP (2010). Mammalian microRNAs predominantly act to decrease target mRNA levels. Nature.

[evu151-B99] Hami D, Grimes AC, Tsai H-J, Kirby ML (2011). Zebrafish cardiac development requires a conserved secondary heart field. Development.

[evu151-B100] Han J, Denli AM, Gage FH (2012). The enemy within: intronic miR-26b represses its host gene, ctdsp2, to regulate neurogenesis. Genes Dev..

[evu151-B101] Han J (2009). Posttranscriptional crossregulation between Drosha and DGCR8. Cell.

[evu151-B102] Han JJ (2004). The Drosha-DGCR8 complex in primary microRNA processing. Genes Dev..

[evu151-B103] Hansen TB (2013). Natural RNA circles function as efficient microRNA sponges. Nature.

[evu151-B104] He XJ, Yan YL, Delaurier A, Postlethwait JH (2011). Observation of miRNA gene expression in zebrafish embryos by *in situ* hybridization to microrna primary transcripts. Zebrafish.

[evu151-B105] Heimberg AM, Sempere LF, Moy VN, Donoghue PCJ, Peterson KJ (2008). MicroRNAs and the advent of vertebrate morphological complexity. Proc Natl Acad Sci U S A..

[evu151-B106] Hendry AP, Berg OK (1999). Secondary sexual characters, energy use, senescence, and the cost of reproduction in sockeye salmon. Can J Zool..

[evu151-B107] Heo I (2009). TUT4 in concert with Lin28 suppresses microRNA biogenesis through pre-microRNA uridylation. Cell.

[evu151-B108] Hertel J (2006). The expansion of the metazoan microRNA repertoire. BMC Genomics.

[evu151-B109] Herzer S, Silahtaroglu A, Meister B (2012). Locked nucleic acid-based in situ hybridisation reveals miR-7a as a hypothalamus-enriched microRNA with a distinct expression pattern. J Neuroendocrinol..

[evu151-B110] Herzog W (2004). Genetic analysis of adenohypophysis formation in zebrafish. Mol Endocrinol..

[evu151-B111] Hoskins LJ, Volkoff H (2012). The comparative endocrinology of feeding in fish: insights and challenges. Gen Comp Endocrinol..

[evu151-B112] Howe K (2013). The zebrafish reference genome sequence and its relationship to the human genome. Nature.

[evu151-B113] Hu W, Coller J (2012). What comes first: translational repression or mRNA degradation? The deepening mystery of microRNA function. Cell Res..

[evu151-B114] Hu Z, Shen WJ, Kraemer FB, Azhar S (2012). MicroRNAs 125a and 455 repress lipoprotein–supported steroidogenesis by targeting scavenger receptor class B type I in steroidogenic cells. Mol Cell Biol..

[evu151-B115] Huang CW (2012). Differential expression patterns of growth-related microRNAs in the skeletal muscle of Nile tilapia (*Oreochromis niloticus*). J Anim Sci..

[evu151-B116] Huang J, Zhao L, Xing L, Chen D (2010). MicroRNA-204 regulates Runx2 protein expression and mesenchymal progenitor cell differentiation. Stem Cells.

[evu151-B117] Huang M-B, Xu H, Xie S-J, Zhou H, Qu L-H (2011). Insulin-like growth factor-1 receptor is regulated by microRNA-133 during skeletal myogenesis. PLoS One.

[evu151-B118] Humphreys DT (2012). Complexity of murine cardiomyocyte mirna biogenesis, sequence variant expression and function. PLoS One.

[evu151-B119] Hwang WY (2013). Efficient genome editing in zebrafish using a CRISPR-Cas system. Nat Biotechnol..

[evu151-B120] Jenny MJ, Aluru N, Hahn ME (2012). Effects of short-term exposure to 2,3,7,8-tetrachlorodibenzo-p-dioxin on microRNA expression in zebrafish embryos. Toxicol Appl Pharmacol..

[evu151-B121] Johnston IA (2006). Environment and plasticity of myogenesis in teleost fish. J Exp Biol..

[evu151-B122] Johnston IA (2009). Embryonic temperature affects muscle fibre recruitment in adult zebrafish: genome-wide changes in gene and microRNA expression associated with the transition from hyperplastic to hypertrophic growth phenotypes. J Exp Biol..

[evu151-B123] Jonsson AC (1991). Regulatory peptides in the pancreas of 2 species of elasmobranchs and in theBrockmann bodies of 4 teleost species. Cell Tissue Res..

[evu151-B124] Jopling C (2010). Zebrafish heart regeneration occurs by cardiomyocyte dedifferentiation and proliferation. Nature.

[evu151-B125] Juanchich A, Le Cam A, Montfort J, Guiguen Y, Bobe J (2013). Identification of differentially expressed miRNAs and their potential targets during fish ovarian development. Biol Reprod..

[evu151-B126] Kang L, Cui X, Zhang Y, Yang C, Jiang Y (2013). Identification of miRNAs associated with sexual maturity in chicken ovary by Illumina small RNA deep sequencing. BMC Genomics.

[evu151-B127] Kapsimali M (2007). MicroRNAs show a wide diversity of expression profiles in the developing and mature central nervous system. Genome Biol..

[evu151-B128] Karsenty G, Wagner EF (2002). Reaching a genetic and molecular understanding of skeletal development. Dev Cell..

[evu151-B129] Katoh T (2009). Selective stabilization of mammalian microRNAs by 3' adenylation mediated by the cytoplasmic poly(A) polymerase GLD-2. Genes Dev..

[evu151-B130] Kawahara Y (2007). Redirection of silencing targets by adenosine-to-inosine editing of miRNAs. Science.

[evu151-B131] Kawahara Y (2008). Frequency and fate of microRNA editing in human brain. Nucleic Acids Res..

[evu151-B132] Kawai S, Amano A (2012). BRCA1 regulates microRNA biogenesis via the DROSHA microprocessor complex. J Cell Biol..

[evu151-B133] Kawakami Y (2006). Wnt/β-catenin signaling regulates vertebrate limb regeneration. Genes Dev..

[evu151-B134] Kawamata T, Seitz H, Tomari Y (2009). Structural determinants of miRNAs for RISC loading and slicer-independent unwinding. Nat Struct Mol Biol..

[evu151-B135] Kedde M (2007). RNA-binding protein Dnd1 inhibits microRNA access to target mRNA. Cell.

[evu151-B136] Kim VN, Han J, Siomi MC (2009). Biogenesis of small RNAs in animals. Nat Rev Mol Cell Biol..

[evu151-B137] Kisliouk T, Meiri N (2013). MiR-138 promotes the migration of cultured chicken embryonic hypothalamic cells by targeting reelin. Neuroscience.

[evu151-B138] Kloosterman WP, Lagendijk AK, Ketting RF, Moulton JD, Plasterk RHA (2007). Targeted inhibition of miRNA maturation with morpholinos reveals a role for miR-375 in pancreatic islet development. PLoS Biol..

[evu151-B139] Knaut H, Werz C, Geisler R, Nüsslein-Volhard C, Tübingen 2000 Screen Consortium (2003). A zebrafish homologue of the chemokine receptor Cxcr4 is a germ-cell guidance receptor. Nature.

[evu151-B140] Köprunner M, Thisse C, Thisse B, Raz E (2001). A zebrafish nanos-related gene is essential for the development of primordial germ cells. Genes Dev..

[evu151-B141] Kosik KS (2013). Molecular biology: circles reshape the RNA world. Nature.

[evu151-B142] Kwak PB, Tomari Y (2012). The N domain of Argonaute drives duplex unwinding during RISC assembly. Nat Struct Mol Biol..

[evu151-B143] Lal A (2009). miR-24 inhibits cell proliferation by targeting E2F2, MYC, and other cell-cycle genes via binding to “seedless” 3' UTR microRNA recognition elements. Mol Cell..

[evu151-B144] Lalwani MK (2012). Reverse genetics screen in zebrafish identifies a role of miR-142a-3p in vascular development and integrity. PLoS One.

[evu151-B145] Landgraf P (2007). A mammalian microRNA expression atlas based on small RNA library sequencing. Cell.

[evu151-B146] Le MTN (2009). MicroRNA-125b is a novel negative regulator of p53. Genes Dev..

[evu151-B147] Leatherland JF (1976). Structure of the nongranulated cells in the hypophyseal rostral pars distalis of cyclostomes and actinopterygians. Cell Tissue Res..

[evu151-B148] Lee EJ (2008). Systematic evaluation of microRNA processing patterns in tissues, cell lines, and tumors. RNA.

[evu151-B149] Lee H-J, Palkovits M, Young WS (2006). miR-7b, a microRNA up-regulated in the hypothalamus after chronic hyperosmolar stimulation, inhibits Fos translation. Proc Natl Acad Sci U S A..

[evu151-B150] Lee LW (2010). Complexity of the microRNA repertoire revealed by next-generation sequencing. RNA.

[evu151-B151] Lee MT (2013). Nanog, Pou5f1 and SoxB1 activate zygotic gene expression during the maternal-to-zygotic transition. Nature.

[evu151-B152] Lee RC, Feinbaum RL, Ambros V (1993). The *C. elegans* heterochronic gene lin-4 encodes small RNAs with antisense complementarity to lin-14. Cell.

[evu151-B153] Lee Y, Jeon K, Lee JT, Kim S, Kim VN (2002). MicroRNA maturation: stepwise processing and subcellular localization. EMBO J..

[evu151-B154] Lee Y (2003). The nuclear RNase III Drosha initiates microRNA processing. Nature.

[evu151-B155] Leone V (2011). A TSH-CREB1-microRNA loop is required for thyroid cell growth. Mol Endocrinol..

[evu151-B156] Leucci E (2013). microRNA-9 targets the long non-coding RNA MALAT1 for degradation in the nucleus. Sci Rep..

[evu151-B157] Leucht C (2008). MicroRNA-9 directs late organizer activity of the midbrain-hindbrain boundary. Nat Neurosci..

[evu151-B158] Leung AKL, Calabrese JM, Sharp PA (2006). Quantitative analysis of Argonaute protein reveals microRNA-dependent localization to stress granules. Proc Natl Acad Sci U S A..

[evu151-B159] Lewis KE (1999). Control of muscle cell-type specification in the zebrafish embryo by Hedgehog signalling. Dev Biol..

[evu151-B160] Li SC (2011). Interrogation of rabbit miRNAs and their isomiRs. Genomics.

[evu151-B161] Li X, Cassidy JJ, Reinke CA, Fischboeck S, Carthew RW (2009). A microRNA imparts robustness against environmental fluctuation during development. Cell.

[evu151-B162] Li X, Ma J, Fang Q, Li Y (2013). Transcription alterations of microRNAs, cytochrome P4501A and 3A, and AhR and PXR in the liver of zebrafish exposed to crude microcystins. Toxicon.

[evu151-B163] Lim LP, Glasner ME, Yekta S, Burge CB, Bartel DP (2003). Vertebrate microRNA genes. Science.

[evu151-B164] Liu N-A (2003). Pituitary corticotroph ontogeny and regulation in transgenic zebrafish. Mol Endocrinol..

[evu151-B165] Liu N-A (2006). Prolactin receptor signaling mediates the osmotic response of embryonic zebrafish lactotrophs. Mol Endocrinol..

[evu151-B166] Liu X, Ning G, Meng A, Wang Q (2012). MicroRNA-206 regulates cell movements during zebrafish gastrulation by targeting prickle1a and regulating c-Jun N-terminal kinase 2 phosphorylation. Mol Cell Biol..

[evu151-B167] Liu Y (2013). Inheritable and precise large genomic deletions of non-coding RNA genes in zebrafish using TALENs. PLoS One.

[evu151-B168] Liu YW, Chan WK (2002). Thyroid hormones are important for embryonic to larval transitory phase in zebrafish. Differentiation.

[evu151-B169] Llorens F (2013). A highly expressed miR-101 isomiR is a functional silencing small RNA. BMC Genomics.

[evu151-B170] Loh Y-HE, Yi SV, Streelman JT (2011). Evolution of microRNAs and the diversification of species. Genome Biol Evol..

[evu151-B171] Lubzens E, Young G, Bobe J, Cerdà J (2010). Oogenesis in teleosts: how fish eggs are formed. Gen Comp Endocrinol..

[evu151-B172] Luciano DJ, Mirsky H, Vendetti NJ, Maas S (2004). RNA editing of a miRNA precursor. RNA.

[evu151-B173] Luo L (2010). Microarray-based approach identifies differentially expressed micrornas in porcine sexually immature and mature testes. PLoS One.

[evu151-B174] Ma H (2012). Characterization of the rainbow trout egg microRNA transcriptome. PLoS One.

[evu151-B175] Macdonald R (1997). The Pax protein Noi is required for commissural axon pathway formation in the rostral forebrain. Development.

[evu151-B176] Melamed ZE (2013). Alternative splicing regulates biogenesis of miRNAs located across exon-intron junctions. Mol Cell..

[evu151-B177] Melo SA (2010). A genetic defect in exportin-5 traps precursor microRNAs in the nucleus of cancer cells. Cancer Cell.

[evu151-B178] Memczak S (2013). Circular RNAs are a large class of animal RNAs with regulatory potency. Nature.

[evu151-B179] Mennigen JA, Skiba-Cassy S, Panserat S (2013). Ontogenesis of expression of metabolic genes and microRNAs in rainbow trout alevins during the transition from the endogenous to the exogenous feeding period. J Exp Biol..

[evu151-B180] Mennigen JA (2012). Postprandial regulation of hepatic microRNAs predicted to target the insulin pathway in rainbow trout. PLoS One.

[evu151-B181] Michlewski G, Guil S, Semple CA, Cáceres JF (2008). Posttranscriptional regulation of miRNAs harboring conserved terminal loops. Mol Cell..

[evu151-B182] Mickoleit M, Banisch TU, Raz E (2011). Regulation of hub mRNA stability and translation by miR430 and the dead end protein promotes preferential expression in zebrafish primordial germ cells. Dev Dyn..

[evu151-B183] Mishima Y (2012). Widespread roles of microRNAs during zebrafish development and beyond. Dev Growth Differ..

[evu151-B184] Mishima Y (2006). Differential regulation of germline mRNAs in soma and germ cells by zebrafish miR-430. Curr Biol..

[evu151-B185] Mishima Y (2009). Zebrafish miR-1 and miR-133 shape muscle gene expression and regulate sarcomeric actin organization. Genes Dev..

[evu151-B186] Moon TW (2001). Glucose intolerance in teleost fish: fact or fiction?. Comp Biochem Physiol B Biochem Mol Biol..

[evu151-B187] Moretti F, Kaiser C, Zdanowicz-Specht A, Hentze MW (2012). PABP and the poly(A) tail augment microRNA repression by facilitated miRISC binding. Nat Struct Mol Biol..

[evu151-B188] Morton SU (2008). microRNA-138 modulates cardiac patterning during embryonic development. Proc Natl Acad Sci U S A..

[evu151-B189] Nagahama Y (1994). Endocrine regulation of gametogenesis in fish. Int J Dev Biol..

[evu151-B190] Nakamura A, Seydoux G (2008). Less is more: specification of the germline by transcriptional repression. Development.

[evu151-B191] Nakamura Y (2012). Sox9 is upstream of microRNA-140 in cartilage. Appl Biochem Biotechnol..

[evu151-B192] Nelson JS (2006). Fishes of the world.

[evu151-B193] Nemoto T, Mano A, Shibasaki T (2012). Increased expression of miR-325-3p by urocortin 2 and its involvement in stress-induced suppression of LH secretion in rat pituitary. Am J Physiol Endocrinol Metab..

[evu151-B194] Nica G (2006). Eya1 is required for lineage-specific differentiation, but not for cell survival in the zebrafish adenohypophysis. Dev Biol..

[evu151-B195] Nicoli S (2012). miR-221 is required for endothelial tip cell behaviors during vascular development. Dev Cell..

[evu151-B196] Niwa R, Slack FJ (2007). The evolution of animal microRNA function. Curr Opin Genet Dev..

[evu151-B197] Ochi H, Westerfield M (2007). Signaling networks that regulate muscle development: lessons from zebrafish. Dev Growth Differ..

[evu151-B198] Okamura K, Hagen JW, Duan H, Tyler DM, Lai EC (2007). The mirtron pathway generates microRNA-class regulatory RNAs in *Drosophila*. Cell.

[evu151-B199] Ørom UA, Nielsen FC, Lund AH (2008). MicroRNA-10a binds the 5′UTR of ribosomal protein mRNAs and enhances their translation. Mol Cell..

[evu151-B200] Papaioannou MD (2011). Loss of Dicer in sertoli cells has a major impact on the testicular proteome of mice. Mol Cell Proteomics..

[evu151-B201] Paroo Z, Ye X, Chen S, Liu Q (2009). Phosphorylation of the human microRNA-generating complex mediates MAPK/Erk signaling. Cell.

[evu151-B202] Pasquinelli AE, Ruvkun G (2002). Control of developmental timing by microRNAs and their targets. Annu Rev Cell Dev Biol..

[evu151-B203] Plaisance V (2006). MicroRNA-9 controls the expression of granuphilin/Slp4 and the secretory response of insulin-producing Cells. J Biol Chem..

[evu151-B204] Plisetskaya EM (1989). Physiology of fish endocrine pancreas. Fish Physiol Biochem..

[evu151-B205] Poss KD, Keating MT, Nechiporuk A (2003). Tales of regeneration in zebrafish. Dev Dyn..

[evu151-B206] Poss KD, Shen J, Keating MT (2000). Induction of lef1 during zebrafish fin regeneration. Dev Dyn..

[evu151-B207] Power DM (2001). Thyroid hormones in growth and development of fish. Comp Biochem Physiol C Toxicol Pharmacol..

[evu151-B208] Poy MN (2004). A pancreatic islet-specific microRNA regulates insulin secretion. Nature.

[evu151-B209] Pullen TJ, Da Silva Xavier G, Kelsey G, Rutter GA (2011). miR-29a and miR-29b contribute to pancreatic β-cell-specific silencing of monocarboxylate transporter 1 (Mct1). Mol Cell Biol..

[evu151-B210] Rakoczy J (2013). MicroRNAs-140-5p/140-3p modulate leydig cell numbers in the developing mouse testis. Biol Reprod..

[evu151-B211] Ramachandra R, Salem M, Gahr S, Rexroad C, Yao J (2008). Cloning and characterization of microRNAs from rainbow trout (*Oncorhynchus mykiss*): their expression during early embryonic development. BMC Dev Biol..

[evu151-B212] Ramachandran R, Fausett BV, Goldman D (2010). Ascl1a regulates Muller glia dedifferentiation and retinal regeneration through a Lin-28-dependent, let-7 microRNA signalling pathway. Nat Cell Biol..

[evu151-B213] Ramalingam P (2014). Biogenesis of intronic miRNAs located in clusters by independent transcription and alternative splicing. RNA.

[evu151-B214] Rand-Weaver M, Baker B, Kawauchi H (1991). Cellular localization of somatolactin in the pars intermedia of some teleost fishes. Cell Tissue Res..

[evu151-B215] Rinn JL, Huarte M (2011). To repress or not to repress: This is the guardian's question. Trends Cell Biol..

[evu151-B216] Rissland OS, Hong S-J, Bartel DP (2011). MicroRNA destabilization enables dynamic regulation of the miR-16 family in response to cell-cycle changes. Mol Cell..

[evu151-B217] Ruby JG, Jan CH, Bartel DP (2007). Intronic microRNA precursors that bypass Drosha processing. Nature.

[evu151-B218] Rüegger S, Großhans H (2012). MicroRNA turnover: when, how, and why. Trends Biochem Sci..

[evu151-B219] Rybak A (2008). A feedback loop comprising lin-28 and let-7 controls pre-let-7 maturation during neural stem-cell commitment. Nat Cell Biol..

[evu151-B220] Salem M, Xiao C, Womack J, Rexroad C, Yao J (2010). A microRNA repertoire for functional genome research in rainbow trout (*Oncorhynchus mykiss*). Mar Biotechnol..

[evu151-B221] Salzman DW, Shubert-Coleman J, Furneaux H (2007). P68 RNA helicase unwinds the human let-7 microRNA precursor duplex and is required for let-7-directed silencing of gene expression. J Biol Chem..

[evu151-B222] Saunders MA, Liang H, Li W-H (2007). Human polymorphism at microRNAs and microRNA target sites. Proc Natl Acad Sci U S A..

[evu151-B223] Sbrogna JL, Barresi MJF, Karlstrom RO (2003). Multiple roles for Hedgehog signaling in zebrafish pituitary development. Dev Biol..

[evu151-B224] Schreibman MP, Leatherland JF, Mckeown BA (1973). Functional morphology of the teleost pituitary gland. Am Zool..

[evu151-B225] Schulz RW (2010). Spermatogenesis in fish. Gen Comp Endocrinol..

[evu151-B226] Shaham O (2013). Pax6 regulates gene expression in the vertebrate lens through miR-204. PLoS Genet..

[evu151-B227] Sharova LV (2009). Database for mRNA half-life of 19 977 genes obtained by DNA microarray analysis of pluripotent and differentiating mouse embryonic stem cells. DNA Res..

[evu151-B228] Shin C (2010). Expanding the microRNA targeting code: functional sites with centered pairing. Mol Cell..

[evu151-B229] Silverstone AM, Hammell L (2002). Spinal deformities in farmed Atlantic salmon. Can Vet J..

[evu151-B230] Soares A (2009). Parallel DNA pyrosequencing unveils new zebrafish microRNAs. BMC Genomics.

[evu151-B231] Soni K (2013). MiR-34 is maternally inherited in *Drosophila melanogaster* and *Danio rerio*. Nucleic Acids Res..

[evu151-B232] Starega-Roslan J (2011). Structural basis of microRNA length variety. Nucleic Acids Res..

[evu151-B233] Staton AA, Knaut H, Giraldez AJ (2011). miRNA regulation of Sdf1 chemokine signaling provides genetic robustness to germ cell migration. Nat Genet..

[evu151-B234] Stebler J (2004). Primordial germ cell migration in the chick and mouse embryo: the role of the chemokine SDF-1/CXCL12. Dev Biol..

[evu151-B235] Subtelny AO, Eichhorn SW, Chen GR, Sive H, Bartel DP (2014). Poly(A)-tail profiling reveals an embryonic switch in translational control. Nature.

[evu151-B236] Sundaram GM (2013). “See-saw” expression of microRNA-198 and FSTL1 from a single transcript in wound healing. Nature.

[evu151-B237] Suzuki HI, Miyazono K (2011). Emerging complexity of microRNA generation cascades. J Biochem..

[evu151-B238] Suzuki HI (2011). MCPIP1 ribonuclease antagonizes Dicer and terminates microRNA biogenesis through precursor microrna degradation. Mol Cell..

[evu151-B239] Sweetman D (2008). Specific requirements of MRFs for the expression of muscle specific microRNAs, miR-1, miR-206 and miR-133. Dev Biol..

[evu151-B240] Tadros W, Lipshitz HD (2009). The maternal-to-zygotic transition: a play in two acts. Development.

[evu151-B241] Takada S, Berezikov E, Choi YL, Yamashita Y, Mano H (2009). Potential role of miR-29b in modulation of Dnmt3a and Dnmt3b expression in primordial germ cells of female mouse embryos. RNA.

[evu151-B242] Tang X, Muniappan L, Tang G, Özcan S (2009). Identification of glucose-regulated miRNAs from pancreatic β cells reveals a role for miR-30d in insulin transcription. RNA.

[evu151-B243] Tang X-L, Xu M-J, Li Z-H, Pan Q, Fu J-H (2013). Effects of vitamin E on expressions of eight microRNAs in the liver of Nile tilapia (*Oreochromis niloticus*). Fish Shellfish Immunol..

[evu151-B244] Tani S, Kusakabe R, Naruse K, Sakamoto H, Inoue K (2010). Genomic organization and embryonic expression of miR-430 in medaka (*Oryzias latipes*): insights into the post-transcriptional gene regulation in early development. Gene.

[evu151-B245] Taranger GL (2010). Control of puberty in farmed fish. Gen Comp Endocrinol..

[evu151-B246] Tarasov V (2007). Differential regulation of microRNAs by p53 revealed by massively parallel sequencing: miR-34a is a p53 target that induces apoptosis and G1-arrest. Cell Cycle.

[evu151-B247] Tarver JE (2013). miRNAs: small genes with big potential in metazoan phylogenetics. Mol Biol Evol..

[evu151-B248] Tay Y, Zhang JQ, Thomson AM, Lim B, Rigoutsos I (2008). MicroRNAs to Nanog, Oct4 and Sox2 coding regions modulate embryonic stem cell differentiation. Nature.

[evu151-B249] Tessmar-Raible K (2007). Conserved sensory-neurosecretory cell types in annelid and fish forebrain: insights into hypothalamus evolution. Cell.

[evu151-B250] Thatcher EJ, Paydar I, Anderson KK, Patton JG (2008). Regulation of zebrafish fin regeneration by microRNAs. Proc Natl Acad Sci U S A..

[evu151-B251] Tomari Y, Zamore PD (2005). MicroRNA biogenesis: drosha can't cut it without a partner. Curr Biol..

[evu151-B252] Trabucchi M (2009). The RNA-binding protein KSRP promotes the biogenesis of a subset of microRNAs. Nature.

[evu151-B253] Treiber T, Treiber N, Meister G (2012). Regulation of microRNA biogenesis and function. Thromb Haemost..

[evu151-B254] Urnov FD, Rebar EJ, Holmes MC, Zhang HS, Gregory PD (2010). Genome editing with engineered zinc finger nucleases. Nat Rev Genet..

[evu151-B255] Venkatarama T (2010). Repression of zygotic gene expression in the *Xenopus* germline. Development.

[evu151-B256] Venkatesh B (2003). Evolution and diversity of fish genomes. Curr Opin Genet Dev..

[evu151-B257] Volkoff H (2005). Neuropeptides and the control of food intake in fish. Gen Comp Endocrinol..

[evu151-B258] Wainwright EN (2013). SOX9 regulates microRNA miR-202-5p/3p expression during mouse testis differentiation. Biol Reprod..

[evu151-B259] Wang X (2011). Prdm1a and miR-499 act sequentially to restrict Sox6 activity to the fast-twitch muscle lineage in the zebrafish embryo. Development.

[evu151-B260] Wei C, Salichos L, Wittgrove CM, Rokas A, Patton JG (2012). Transcriptome-wide analysis of small RNA expression in early zebrafish development. RNA.

[evu151-B261] Weidinger G, Grotek B, Wehner D (2013). Notch signaling coordinates cellular proliferation with differentiation during zebrafish fin regeneration. Development.

[evu151-B262] Weidinger G (2003). Dead end, a novel vertebrate germ plasm component, is required for zebrafish primordial germ cell migration and survival. Curr Biol..

[evu151-B263] Wienholds E, Koudijs MJ, Van Eeden FJM, Cuppen E, Plasterk RHA (2003). The microRNA-producing enzyme Dicer1 is essential for zebrafish development. Nat Genet..

[evu151-B264] Wienholds E (2005). MicroRNA expression in zebrafish embryonic development. Science.

[evu151-B265] Wightman B, Ha I, Ruvkun G (1993). Posttranscriptional regulation of the heterochronic gene Lin-14 by Lin-4 mediates temporal pattern-formation in *C. elegans*. Cell.

[evu151-B266] Winter J, Jung S, Keller S, Gregory RI, Diederichs S (2009). Many roads to maturity: microRNA biogenesis pathways and their regulation. Nat Cell Biol..

[evu151-B267] Wolf K, Quimby MC (1962). Established eurythermic line of fish cells in vitro. Science.

[evu151-B268] Woltering JM, Durston AJ (2008). MiR-10 represses HoxB1a and HoxB3a in zebrafish. PLoS One.

[evu151-B269] Wu H (2010). A splicing-independent function of SF2/ASF in microRNA processing. Mol Cell..

[evu151-B270] Wu Y, Koenig RJ (2000). Gene regulation by thyroid hormone. Trends Endocrinol Metab..

[evu151-B271] Wyman SK (2011). Post-transcriptional generation of miRNA variants by multiple nucleotidyl transferases contributes to miRNA transcriptome complexity. Genome Res..

[evu151-B272] Xia JH, He XP, Bai ZY, Yue GH (2011). Identification and characterization of 63 microRNAs in the Asian seabass *Lates calcarifer*. PLoS One.

[evu151-B273] Xiao J (2014). Identification and characterization of microRNAs in ovary and testis of Nile Tilapia (*Oreochromis niloticus*) by using solexa sequencing technology. PLoS One.

[evu151-B274] Xu Z (2013). Identification and characterization of microRNAs in channel catfish (*Ictalurus punctatus*) by using solexa sequencing technology. PLoS One.

[evu151-B275] Yadav RP, Kotaja N (2014). Small RNAs in spermatogenesis. Mol Cell Endocrinol..

[evu151-B276] Yamagata K (2009). Maturation of microRNA is hormonally regulated by a nuclear receptor. Mol Cell..

[evu151-B277] Yamano K, Miwa S, Obinata T, Inui Y (1991). Thyroid hormone regulates developmental changes in muscle during flounder metamorphosis. Gen Comp Endocrinol..

[evu151-B278] Yan B, Guo J-T, Zhao L-H, Zhao J-L (2012a). microRNA expression signature in skeletal muscle of Nile tilapia. Aquaculture.

[evu151-B279] Yan B, Guo J-T, Zhao L-H, Zhao J-L (2012b). MiR-30c: a novel regulator of salt tolerance in tilapia. Biochem Biophys Res Commun..

[evu151-B280] Yan B, Guo JT, Zhu CD, Zhao LH, Zhao JL (2013). miR-203b: a novel regulator of MyoD expression in tilapia skeletal muscle. J Exp Biol..

[evu151-B281] Yan B, Zhao L-H, Guo J-T, Zhao J-L (2012). miR-429 regulation of osmotic stress transcription factor 1 (OSTF1) in tilapia during osmotic stress. Biochem Biophys Res Commun..

[evu151-B282] Yan B, Zhu C-D, Guo J-T, Zhao L-H, Zhao J-L (2013). miR-206 regulates the growth of the teleost tilapia (*Oreochromis niloticus*) through the modulation of IGF-1 gene expression. J Exp Biol..

[evu151-B283] Yan HL (2009). Repression of the miR-17-92 cluster by p53 has an important function in hypoxia-induced apoptosis. EMBO J..

[evu151-B284] Yan XC (2012). Identification and profiling of microRNAs from skeletal muscle of the common carp. PLoS One.

[evu151-B285] Yan Y-L (2002). A zebrafish sox9 gene required for cartilage morphogenesis. Development.

[evu151-B286] Yang R, Dai Z, Chen S, Chen L (2011). MicroRNA-mediated gene regulation plays a minor role in the transcriptomic plasticity of cold-acclimated Zebrafish brain tissue. BMC Genomics.

[evu151-B287] Yang W (2006). Modulation of microRNA processing and expression through RNA editing by ADAR deaminases. Nat Struct Mol Biol..

[evu151-B288] Yang X (2012). Differentially expressed plasma microRNAs in premature ovarian failure patients and the potential regulatory function of mir-23a in granulosa cell apoptosis. Reproduction.

[evu151-B289] Yang XJ (2009). miR-449a and miR-449b are direct transcriptional targets of E2F1 and negatively regulate pRb-E2F1 activity through a feedback loop by targeting CDK6 and CDC25A. Genes Dev..

[evu151-B290] Yi R, Qin Y, Macara IG, Cullen BR (2003). Exportin-5 mediates the nuclear export of pre-microRNAs and short hairpin RNAs. Genes Dev..

[evu151-B291] Yi S (2013). Identification and characterization of microRNAs involved in growth of blunt snout bream (*Megalobrama amblycephala*) by solexa sequencing. BMC Genomics.

[evu151-B292] Yin M (2012). Transactivation of microRNA-383 by steroidogenic factor-1 promotes estradiol release from mouse ovarian granulosa cells by targeting RBMS1. Mol Endocrinol..

[evu151-B293] Yin VP, Lepilina A, Smith A, Poss KD (2012). Regulation of zebrafish heart regeneration by miR-133. Dev Biol..

[evu151-B294] Yin VP (2008). Fgf-dependent depletion of microRNA-133 promotes appendage regeneration in zebrafish. Genes Dev..

[evu151-B295] Yu D (2010). miR-451 protects against erythroid oxidant stress by repressing 14-3-3ζ. Genes Dev..

[evu151-B296] Yu YM (2011). MicroRNA miR-133b is essential for functional recovery after spinal cord injury in adult zebrafish. Eur J Neurosci..

[evu151-B297] Zeng Y, Cullen BR (2004). Structural requirements for pre-microRNA binding and nuclear export by exportin 5. Nucleic Acids Res..

[evu151-B298] Zhang L (2011). MicroRNA expression changes during zebrafish development induced by perfluorooctane sulfonate. J Appl Toxicol..

[evu151-B299] Zhang N (2013a). MicroRNA 375 mediates the signaling pathway of corticotropin-releasing factor (CRF) regulating pro-opiomelanocortin (POMC) expression by targeting mitogen-activated protein kinase 8. J Biol Chem..

[evu151-B300] Zhang Q (2013b). MicroRNA-181a suppresses mouse granulosa cell proliferation by targeting activin receptor IIA. PLoS One.

[evu151-B301] Zhang R (2013c). In vivo cardiac reprogramming contributes to zebrafish heart regeneration. Nature.

[evu151-B302] Zhang Z, Florez S, Gutierrez-Hartmann A, Martin JF, Amendt BA (2010). MicroRNAs regulate pituitary development, and microRNA 26b specifically targets lymphoid enhancer factor 1 (Lef-1), which modulates pituitary transcription factor 1 (Pit-1) expression. J Biol Chem..

[evu151-B303] Zhao ZY (2010). A negative regulatory loop between microRNA and Hox gene controls posterior identities in *Caenorhabditis elegans*. PLoS Genet..

[evu151-B304] Zhu Q-H (2008). A diverse set of microRNAs and microRNA-like small RNAs in developing rice grains. Genome Res..

[evu151-B305] Zhu Y-P (2012). Identification of common carp (*Cyprinus carpio*) microRNAs and microRNA-related SNPs. BMC Genomics.

[evu151-B306] Ziebarth JD, Bhattacharya A, Chen A, Cui Y (2012). PolymiRTS database 2.0: linking polymorphisms in microRNA target sites with human diseases and complex traits. Nucleic Acids Res..

[evu151-B307] Zisoulis DG, Kai ZS, Chang RK, Pasquinelli AE (2012). Autoregulation of microRNA biogenesis by let-7 and Argonaute. Nature.

